# Tagger—A Swiss army knife for multiomics to dissect cell type–specific mechanisms of gene expression in mice

**DOI:** 10.1371/journal.pbio.3000374

**Published:** 2019-08-08

**Authors:** Lech Kaczmarczyk, Vikas Bansal, Ashish Rajput, Raza-ur Rahman, Wiesław Krzyżak, Joachim Degen, Stefanie Poll, Martin Fuhrmann, Stefan Bonn, Walker Scot Jackson

**Affiliations:** 1 Wallenberg Center for Molecular Medicine, Department of Clinical and Experimental Medicine, Linköping University, Linköping, Sweden; 2 German Center for Neurodegenerative Diseases, Bonn, Germany; 3 Institute for Medical Systems Biology, Center for Molecular Neuroscience, University Medical Center Hamburg-Eppendorf, Hamburg, Germany; 4 Life & Medical Sciences Institute, University of Bonn, Bonn, Germany; 5 German Center for Neurodegenerative Diseases, Tübingen, Germany; Yale University, UNITED STATES

## Abstract

A deep understanding of how regulation of the multiple levels of gene expression in mammalian tissues give rise to complex phenotypes has been impeded by cellular diversity. A handful of techniques were developed to tag-select nucleic acids of interest in specific cell types, thereby enabling their capture. We expanded this strategy by developing the Tagger knock-in mouse line bearing a quad-cistronic transgene combining enrichment tools for nuclei, nascent RNA, translating mRNA, and mature microRNA (miRNA). We demonstrate that Tagger can capture the desired nucleic acids, enabling multiple omics approaches to be applied to specific cell types in vivo using a single transgenic mouse line.

## Abstract 2

This Methods and Resources paper describes Tagger, a knock-in mouse line bearing a quad-cistronic transgene that enables the capture of translating mRNAs, mature miRNAs, pulse-labeled total RNA, and the nucleus, all from specific cells of complex tissues.

## Introduction

Gene expression, a compilation of processes actuating information encoded in the genome, is exquisitely controlled at multiple levels [1,2]. In the nucleus, chemical and conformational modifications to chromatin modulate the access of transcriptional machineries to gene regulatory elements in DNA. A coordinated action of activators and repressors, as well as of chromatin-modifying enzymes, govern which information is converted into RNA transcripts and which remains silent. Some transcripts are then processed into mature mRNAs and shuttled to the cytoplasm, where the encoded information is translated by ribosomes into protein. Translation is controlled at both the level of ribosome subunits binding to mRNAs and through the actions of the RNA-induced silencing complex (RISC) guided by microRNAs (miRNAs) [3,4]. The latter can stall translation or target mRNAs for degradation [4,5]. This landscape is complemented by various classes of long noncoding RNAs (lncRNAs), RNA species incompletely understood but thought to have a role in many aspects of gene expression regulation [6,7].

Our understanding of how gene expression is coordinated at the cellular level of mammalian tissues has been impeded by their heterogenous nature. Typical assays, when applied to such tissues, provide information on general regulatory trends but give little to no information about the cellular source of the observed changes, or about the specific level(s) of gene expression regulation that directed them. An innovative solution to tackle this challenge is the purification of nucleic acids from a related subset of cells after tissue sample lysis. Several strategies were developed that share the common theme of using cell type–specific genetically engineered handles or tags to purify the target nucleic acids [8]. These methods often employ a recombinase system, providing access to a wide spectrum of cell types based on cell identity, developmental stage, or neuronal activity, for example. Transgenic techniques based on this concept have been applied in laboratory mice to purify translating mRNA [9, 10], mature miRNA [11,12], pulse-labeled total RNA [13,14], and chromatin and nuclear RNA [15–17]. These reports demonstrate that each level of gene regulation could be captured from specific cell types with negligible effect of the methods themselves on gene expression.

However, each of these methods alone captures an incomplete picture of gene expression. Hitherto, only a single method was specifically developed to capture more than one regulatory level simultaneously by employing a combination of nuclear and ribosomal tags [18]. We thought more could be gained by combining additional techniques into a single mouse to investigate a fuller spectrum of gene expression simultaneously.

**Fig 1:**
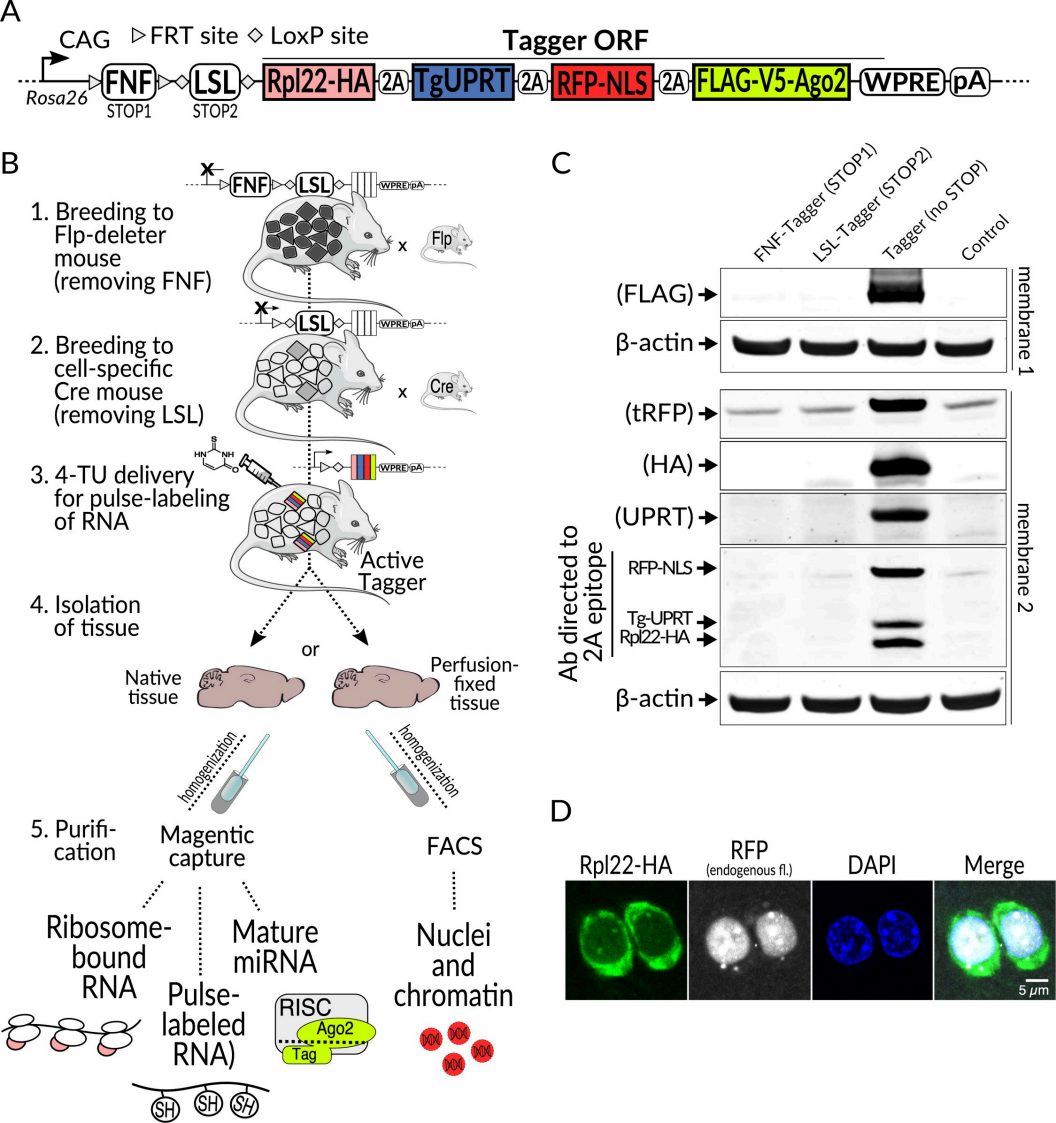
Overview of the Tagger system and validation of expression. (**A**) Schematic of the Rosa26 knock-in Tagger transgene. From left to right: CAG—ubiquitous synthetic CAG promoter; Frt-NeoR-Frt (FNF) and LoxP-STOP-LoxP (LSL) transcriptional STOP cassettes flanked by sites for specific recombinases (Flp and Cre, respectively), allowing cell type–specific, intersectional activation of expression; single ORF encoding four proteins (in colors) separated by 2A peptides:—hemagglutinin-tagged large subunit ribosome protein 22 (Rpl22-HA, Ribo-Tag), *Toxoplasma gondii* Uracil phosphoribosyltransferase (TgUPRT, TU-Tag), red fluorescent protein with three NLSs (RFP-NLS, Nuc-Tag), and FLAG- and V5-tagged Argonaute2 protein (FLAG-V5-Ago2, Ago-Tag); WPRE; pA. (**B**) Example experimental workflow. After transgene activation by breeding to (1) Flp and (2) Cre mice, 4-Thiouracil is injected subcutaneously for metabolic RNA labeling (3); alternatively, breeding to Cre and Flp double transgenic mice would enable intersectional activation by both recombinases. Tissue is then isolated (4), homogenized, and subjected to enrichment of choice (5): affinity purification(s) or FACS of nuclei after optional perfusion-fixation. (**C**) Immunoblot validation of separation of all four Tagger components. Top, individual components are detected with antibodies against specific epitopes, labeled on the left. Bottom, detection with a 2A-specific antibody reveals comparable expression levels for the three components with the residual 2A epitope. Probing was done with the same samples at equal loading amounts on two different membranes. (**D**) Immunofluorescence staining of brain cryosection from a vGluT2-Tagger mouse showing separation of tagger components Rpl22-HA (stained with anti-HA antibody) and RFP-NLS (endogenous fluorescence). The section was counterstained with DAPI. Ago2, Argonaute 2; CAG, cytomegalovirus:chicken actin fusion promoter; Cre, causes recombination; FACS, fluorescence activated cell sorting; Flp, flippase; FNF, Frt-NeoR-Frt; Frt, flippase recognition target; LSL, LoxP-STOP-LoxP; NeoR, neomycin resistance; NLS, nuclear localization signal; ORF, open reading frame; pA, polyadenylation signal; RFP, red fluorescent protein; Rpl22-HA, hemagglutinin-tagged large subunit ribosomal protein 22; tRFP, turbo red fluorescent protein; vGluT2, vesicular glutamate transporter 2; WPRE, Woodchuck Hepatitis Virus Postranscriptional Response Element.

This prompted us to develop Tagger—a causes recombination (Cre)- and/or flippase (Flp)-dependent mouse line that stoichiometrically expresses four proteins for the capture of distinct populations of nucleic acids (Fig 1A and 1B). Tagger captures whole nuclei for chromatin and nuclear RNA (Nuc-Tag), nascent RNA pulse labeled with 4-Thiouracil (TU-Tag), translating mRNA embedded in ribosomes (Ribo-Tag), and mature miRNA bound to Argonaute2 (Ago-Tag) (Fig 1A). Following activation with cell type–specific Cre and/or Flp driver lines, Tagger provides a platform to perform multiple omics experiments on specific cell populations in vivo using the same mouse (Fig 1B). In this report, we describe the development of Tagger and demonstrate the robustness of each component, with comparisons to the original cutting-edge methods that inspired us to create this multifunctional tool.

## Results

### Tagger expression is tightly controlled and specific

Our overall strategy was to co-express four proteins to carry out the four functions. Ribo-Tag functionality was created by expressing the large subunit ribosomal protein 22 (Rpl22) with a hemagglutinin (HA) epitope Tag (Rpl22-HA), TU-Tag functionality via expression of uracil phosphoribosyltransferase from *Toxoplasma gondii* (TgUPRT), Nuc-Tag functionality via expression of a red fluorescent protein (RFP) targeted to the nucleus with a nuclear localization signal (NLS, together RFP-NLS), and Ago-Tag via expression of Argonaute 2 (Ago2) carrying V5 and FLAG epitopes (FLAG-V5-Ago2). All four components were encoded by the same open reading frame and separated by 2A “self-cleaving” peptides [19,20] (Fig 1A and S1A Fig). We gene-targeted the Tagger construct to the ROSA-26 “safe harbor” locus to access a wide variety of cell types [21]. Expression is controlled by two terminators: Frt-NeoR-Frt (FNF) and LoxP-STOP-LoxP (LSL), to enable transgene activation at the intersection of cell populations expressing Flp and Cre driven by distinct promoters, to target cells based on combinations of cell identity markers or activity [22]. A potential problem with this design is the “leakiness” of STOP cassettes. To rule it out, we tested the functionality of the STOP cassettes by crossing Tagger mice with mice expressing Cre or Flp ubiquitously (deleter mice). No detectable expression of any component was observed when either LSL or FNF was present (Fig 1C). In contrast, removal of both led to robust expression in the brain and other organs (Fig 1C, S2A, S2B and S2C Fig). This demonstrates the functionality of both STOP cassettes and thus their suitability for intersectional experiments using both recombinases.

**Fig 2:**
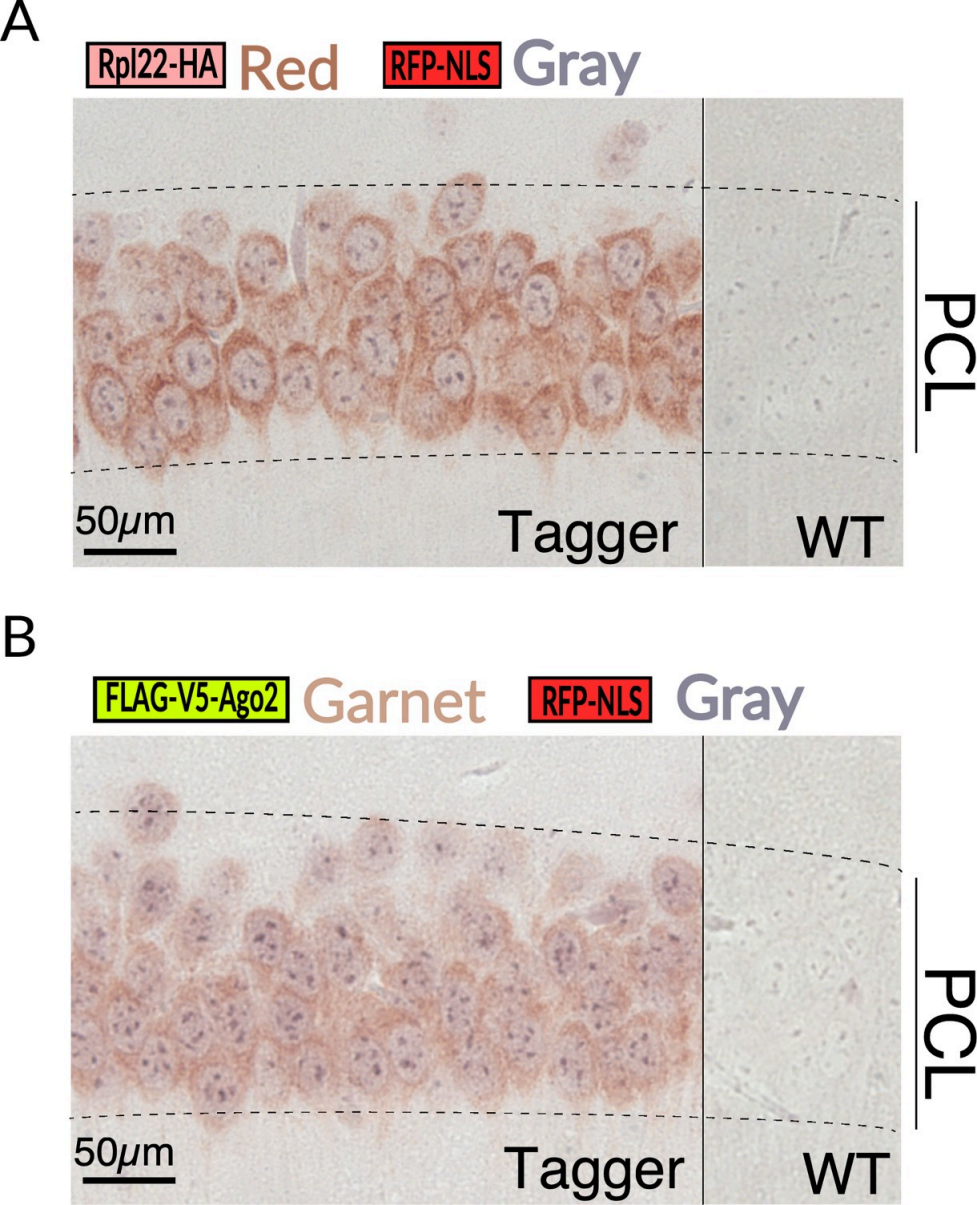
Immunohistochemical validation of subcellular localization. Paraffin sections double-immunostained for Rpl22 (HA) and RFP-NLS (**A**) and Argonaute2 (V5) and RFP-NLS (**B**) confirming separation of Tagger components. HA, hemagglutinin; RFP-NLS, red fluorescent protein-nuclear localization signal; PCL, principal cell layer of the hippocampus; Rpl22, large subunit ribosomal protein 22; WT, wild-type.

Another serious concern was that translation processivity might be impaired. The inclusion of 2A peptide sequences for co-expression had not been previously employed in mice to express either four proteins with distinct functions and cellular localization, or such a large collective molecular weight of protein components (approximately 180 kDa in total). A recent study highlights potential perils and pitfalls with such an approach [23]. However, we did not detect any immunoblot signals corresponding to unseparated components (Fig 1C). Confocal microscopy revealed a clear spatial separation of Rpl22-HA (the first component in the open reading frame [ORF]) and RFP-NLS (the third component) (Fig 1D). Incidentally, in vivo multiphoton imaging of RFP-NLS showed strong fluorescence restricted to cell nuclei, highlighting its potential for additional uses (S3A Fig). To further verify that the components were separated, we co-stained formalin fixed paraffin embedded (FFPE) brain sections with antibodies against the nuclear component RFP-NLS and either Rpl22-HA or FLAG-V5-Ago2 (the last component), both of which should be mostly cytoplasmic. Indeed, we observed RFP-NLS protein restricted to the nucleus, whereas Rpl22-HA and FLAG-V5-Ago2 localized primarily in the cytoplasm (Fig 2A and 2B). Immunoblots probed with an antibody targeting the residual 2A peptides indicated the proteins were both separated and expressed at similar levels (Fig 1C, bottom panel). Furthermore, the protein expression levels of Rpl22-HA protein in ubiquitously activated Tagger and the original RiboTag mice were comparable (S2D Fig). Importantly, mice activated for ubiquitous expression were viable, fertile, showed no obvious phenotypic deficits, and lived to at least 24 months. These results indicate that the transgene expression is tightly controlled, its gene products are synthesized properly, and ubiquitous expression of the four proteins, including modified variants of Rpl22 and Ago2 that hold crucial cellular functions in their native state, is not overtly toxic.

We next sought to determine if Tagger expression could be directed to specific cell types. We first established a Tagger line with the FNF STOP cassette already removed (LSL-Tagger) and then bred it to mice expressing Cre in specific types of neurons. We employed three different Cre drivers: one which demonstrates activity in most glutamatergic (excitatory) neurons throughout the brain, vesicular glutamate transporter 2 (vGluT2)-Cre [24], one to study GABAergic (inhibitory) neurons, glutamic acid decarboxylase 2 (Gad2)-Cre [25], and one for a subset of GABAergic neurons, parvalbumin (PV)-Cre [26]. GABAergic neurons are especially interesting, as they fine-tune excitatory neurons. The Tagger transgene was then activated by crossing LSL-Tagger mice and Cre mice, yielding vGluT2-Tagger, Gad2-Tagger, and PV-Tagger mice, respectively. Immunofluorescent stainings of the cell type–specific markers special AT-rich sequence-binding protein 2 (Satb2), glutamic acid decarboxylase 67 (Gad67), and PV (specific for glutamatergic, GABAergic, and PV neurons, respectively) revealed a high specificity of Tagger expression (determined through HA staining of Rpl22-HA and native fluorescence of RFP-NLS, S2E Fig). These results revealed that the expression of Tagger can be directed to desired cell types.

### Translating mRNAs and mature miRNAs can be purified from the same supernatant

**Fig 3:**
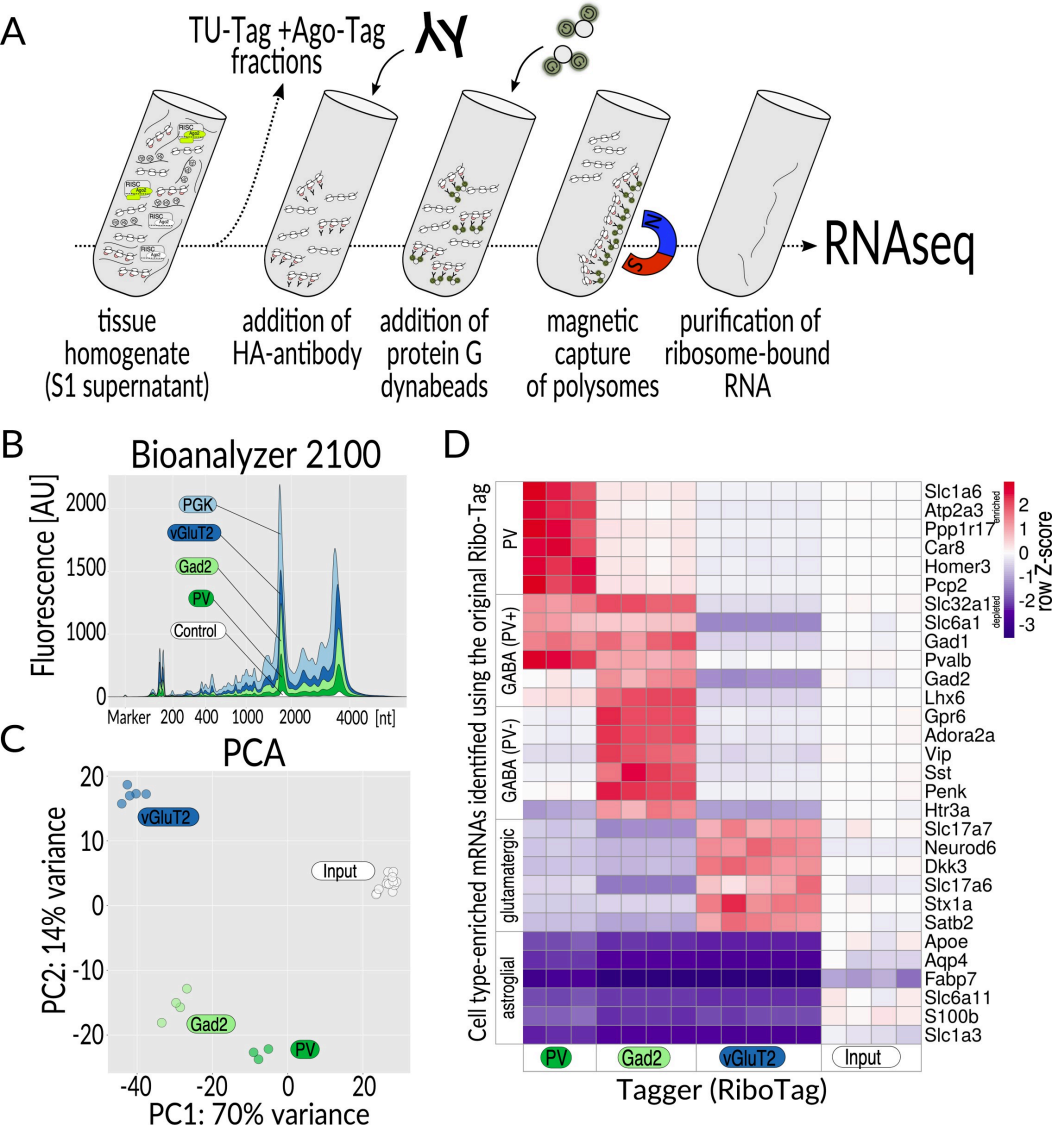
Ribo-Tag. (**A**) Overview of the procedure. Tissue homogenate, following removal of nuclei and cell debris (S1 supernatant), is split into fractions for purifying specific classes of nucleic acids. To the fraction for Ribo-Tag, antibodies directed to the HA epitope are added, and the antigen-antibody complexes containing tagged ribosomes are enriched using protein G dynabeads. Eventually, RNA is released from protein G dynabeads, purified, and subjected to RNAseq. (**B**) Agilent bioanalyzer profiles of cell type–specific mRNA. Relative amounts of RNA correspond to the proportion of the analyzed cell population in the brain (color coded), determined by the Cre driver mouse line that was used (balloon labels, control = no Cre). (**C**) PCA of the data from three analyzed cell populations and input (S1 supernatant). (**D**) Heatmap showing relative distribution of Ribo-Tag (Tagger) TPM values for genes for cell type–enriched mRNAs selected based on data obtained with the original RiboTag mouse [10]. Each column represents one biological replicate. Z-score for each row was calculated to set the input levels to 0: Z = (x–mean(input))/SD(row), where SD is standard deviation. The complete set of TPM values on which panel D is based can be found in S1 Data. AU, arbitrary unit; Cre, causes recombination; HA, hemagglutinin; PCA, principal component analysis; RNAseq, RNA sequencing; TPM, transcripts per million.

We were especially motivated to acquire multiomics data from the same biological samples, as that would allow correlation of changes within individual mice. To this end, we established purification procedures with elements that could be applied to multiple Tagger domains at the same time. For example, when performing the Ribo-Tag procedure, aliquots of the same supernatant may be used for TU-Tag and Ago-Tag modalities (Fig 3A). Achieving such compatibility between different procedures was technically challenging, and only possible to evaluate using *in vivo* material. To assess cell type specificity of Ribo-Tag and Ago-Tag, we tested samples from vGluT2-, Gad2-, and PV-Tagger mice. To limit the number of mice required to establish these protocols, we analyzed whole brain hemispheres.

We observed that the amount of RNAs captured with Ribo-Tag corresponded to the proportion of tagged cells in the brain (Fig 3B). To validate the Ribo-Tag specificity in Tagger mice, we performed RNA sequencing (RNAseq) on the enriched mRNAs and the corresponding input supernatants (the latter were used as reference). Principal component analysis (PCA) showed tight clustering of samples reflecting their cell type of origin (Fig 3C). We then calculated, using DESeq2 [27], differential gene expression between cell type–specific samples and total (input) RNA and examined a list of cell type marker mRNAs created from data we obtained with the original RiboTag mice and largely composed of established cell type markers (S1 Table). Ribo-Tag data from Tagger mice matched the expected enrichments and depletions for all genes on this list, indicating that Ribo-Tag purifications perform comparably well in Tagger as in the original line (Fig 3D).

**Fig 4:**
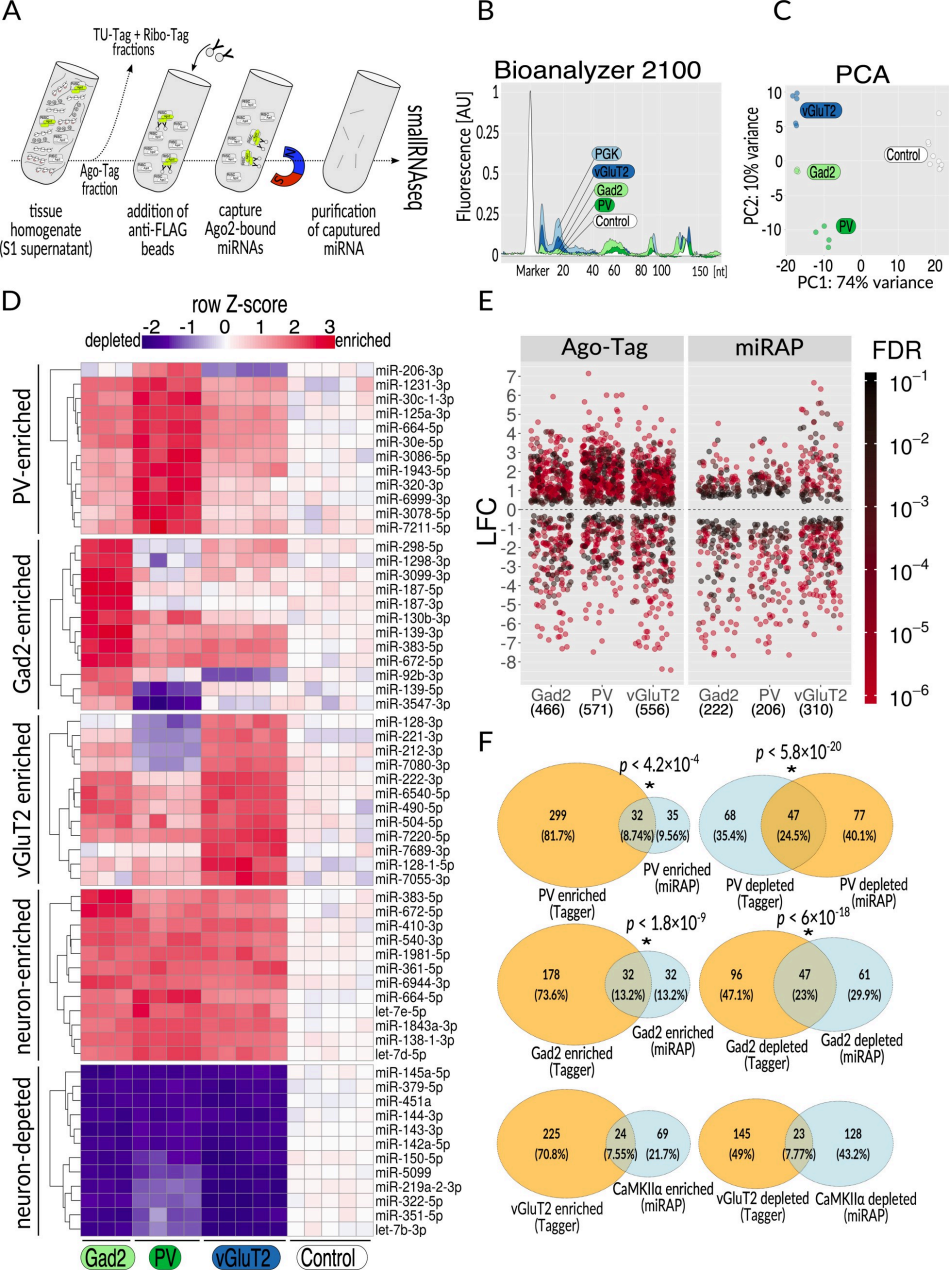
Ago-Tag. (**A**) Overview of the procedure. Tissue homogenate, following removal of nuclei and cell debris (S1 supernatant), is split into fractions for purifying specific classes of nucleic acids. The Ago-Tag fraction is then mixed with anti-FLAG epitope magnetic beads to enrich for Ago-Tag bound to mature miRNAs. The latter are then purified and subjected to small RNAseq. (**B**) Agilent bioanalyzer profiles of Ago-Tag–purified miRNA. Relative amounts of RNA correspond to the proportion of the analyzed cell population in the brain (color coded), determined by the Cre line that was used (balloon labels, control = no Cre). (**C**) PCA of the data from three analyzed cell populations and input (S1 supernatant). (**D**) Heatmap showing relative distribution of VST-normalized counts (calculated using DEseq2 package) for 60 miRNAs assigned to one of five groups: PV enriched, Gad2 enriched, vGluT2 enriched, neuron enriched, and neuron depleted. miRNAs for each group were chosen on the basis of the rank metric (see Methods), by taking the top 12 genes with |LFC| > 1 and FDR < 0.1 and according to the ranking formula (left side of respective heatmap). Z-score for each row was calculated as Z = (x–mean(input))/SD(row), where SD is standard deviation. The complete set of TPM values on which panel D is based can be found in S1 Data. (**E**) Comparison of distribution of significantly (|LFC| > 1, FDR < 0.1) changed miRNAs in Ago-Tag and miRAP [11]; numbers in parentheses denote the number of changed miRNAs. (**F**) Overlap of significantly enriched (LFC > 1, FDR < 0.1) and significantly depleted (LFC > 1, FDR < 0.1) miRNAs between Ago-Tag data and miRAP [11] data. Asterisks denote statistically significant overlaps (hypergeometric test, see also S3 Table). Note the lack of statistical significance for comparisons of data obtained with different Cre drivers (vGluT2-Cre versus CaMKIIα-Cre). Ago2, Argonaute 2; AU, arbitrary unit; CaMKIIα, calcium/calmodulin dependent protein kinase II; Cre, causes recombination; FDR, false discovery rate; Gad2, glutamic acid decarboxylase 2; LFC, Log_2_ fold change; miRAP, miRNA affinity purification; miRNA, microRNA; PCA, principal component analysis; PV, parvalbumin; RNAseq, RNA sequencing; TPM, transcripts per million; vGluT2, vesicular glutamate transporter 2; VST, variance stabilizing transformation.

Having established that the Ribo-Tag component of Tagger yields cell type–specific mRNAs and thus is expressed in the intended cell types, we analyzed cell type–specific miRNAs from vGluT2, Gad2, and PV neurons using the same supernatant (S1 hereafter) as for the Ribo-Tag method (Fig 4A). To this end, we first determined that the lysis buffer for Ribo-Tag (Polysome Buffer [PSB]) can be used to immunoprecipitate (IP) RISC-associated miRNAs using antibodies directed to the FLAG or V5 epitopes on the FLAG-V5-Ago2 component of the Tagger (S4A Fig). Moreover, anti-FLAG antibody directly coupled to magnetic agarose performed comparably to tested configurations of antibodies captured with protein G magnetic beads, as measured by the magnitude of miRNA peaks on Agilent bioanalyzer 2100 profiles (S4A Fig). We also observed that increasing salt concentration has negligible effect on Ribo-Tag efficiency, and therefore we increased the ionic strength by adding extra 300 mM NaCl to the original wash buffer [10] (S4B Fig). After establishing the IP conditions, we positively verified that both, Dicer and endogenous Ago2, co-IP with Ago-Tag and that small subunit ribosomal protein 6 (Rps6) co-IP with Ribo-Tag (S4C Fig). These results indicate that we captured an assembled RISC and translating ribosomes containing both subunits. We also detected wild-type (WT) Ago2 and FLAG-V5-Ago2 from HA IPs, suggesting that at least some of the ribosomes we captured were stalled by RISC (S4C Fig). Thus, Ago-Tag IPs are robust and the flexibility in antibody-bead configurations may, if needed, enable alternative purification strategies.

Akin to Ribo-Tag, the amounts of RNA captured with Ago-Tag corresponded to the proportion of cells of origin in the brain (Fig 4B). After performing small RNAseq and analysis using Oasis 2 [28], the PCA clusters matched with the cell types of origin (Fig 4C). Interestingly, Ago-Tag preparations from vGluT2-, Gad2-, and PV-Tagger samples contained many fewer non-miRNA reads (0.11%, 0.18%, and 0.37%, respectively) than did the corresponding total small RNA preparations (3.8%), indicating a 10–30-fold removal of unbound small RNAs (S2 Table). We quantified miRNA enrichment or depletion levels by comparing the Ago-Tag samples with the input (total brain cytosolic fraction) using DESeq2 [27], like we did for Ribo-Tag. Then, we ranked miRNA expression in specific cell types and prepared lists of miRNAs that were (i) enriched in all, (ii) enriched in only one or (iii) depleted in all three of the analyzed neuron types (Fig 4D). We found some miRNAs depleted from all neurons, some enriched in all neurons, and some specifically enriched in neuronal subtypes, such as miR206-3P for PV, miR139-5p for Gad2, and miR128-3p for vGluT2.

To compare the performance of Ago-Tag with another brain miRNA affinity purification (miRAP) technology [11], we reanalyzed raw miRAP data using Oasis 2. It is important to note that He and colleagues analyzed neocortex, while we analyzed the whole brain. For both data sets, we compared fold changes of cell type–specific miRNAs with respect to corresponding inputs (whole brain miRNAs and neocortical miRNAs in case of Ago-Tag and miRAP, respectively). Especially due to the latter discrepancy, we expected the Ago-Tag samples to show fewer differentially expressed miRNAs, as greater cellular heterogeneity of the tissue would likely obscure the differences between the cell populations in different brain regions. However, in all studied cell types, Ago-Tag revealed more significant changes as compared with miRAP (Fig 4E). Enriched and depleted sets of genes revealed by both methods partially but significantly overlapped (Fig 4F), demonstrating that the specificity and functionality of Ago-Tag is comparable to that of miRAP.

### TU-tagging and purification of thiolated RNA

Incorporation of 4-thiouridinemonophosphate (the active derivative of 4-thiouracil [4-TU]) into nascent RNA is becoming an invaluable technique to study RNA kinetics in cultured cell experiments, and recently proved critical to distinguish between primary transcriptional changes from secondary effects in response to manipulation of cancer pathways [29].

We incorporated cell type–specific 4-TU labeling of nascent RNA in mice (TU-tagging) into the array of Tagger modalities to facilitate studies of transcription kinetics, as well as for analyses of noncoding RNA networks, examples of which were reported in the mouse brain [30,31]. However, several challenges have limited the application of TU-Tagging in mice. One major hurdle is that little is known about 4-TU uptake and removal kinetics in the brain and therefore optimal labeling times. Another challenge is that, even with optimized labeling periods, a low signal is expected due to the competition of 4-TU and endogenous (unlabeled) uracil. Moreover, in vivo experiments involve an inherent fraction of non-labeled cells and, thus, accompany a background signal due to alternative endogenous enzymes also activating 4-TU. Adding to the low expected signal with the high expected background, the purification chemistries are still imperfect, even for cultured cell experiments. Finally, the most descriptive mouse TU-Tagging protocols from which to base our pilot experiments were established for brain endothelial cells [13,14], whereas we aimed to study mature neurons residing on the opposite side of the blood-brain barrier.

**Fig 5:**
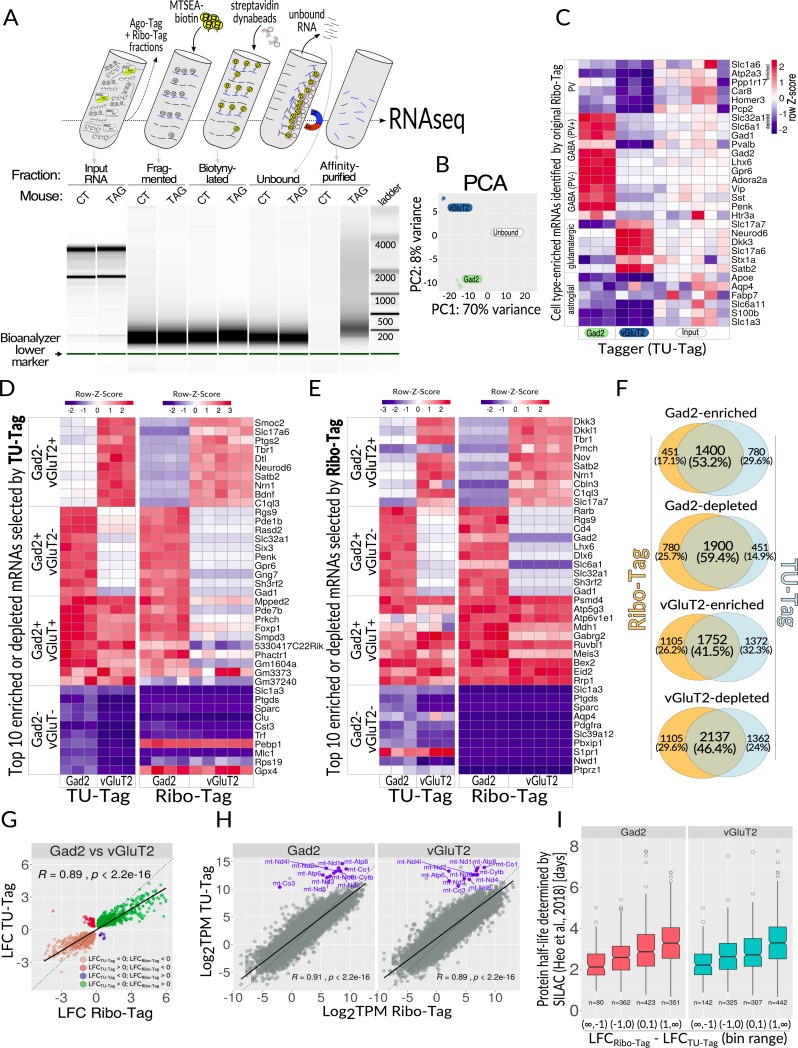
TU-Tag. (**A**) Top, overview of key steps of the procedure. Tissue homogenate, following removal of nuclei and cell debris (S1 supernatant), is split into fractions for purifying specific classes of nucleic acids. Thiolated RNA from the TU-Tag is fragmented, biotinylated, and affinity purified using streptavidin-coated dynabeads, and eventually sequenced. Bottom, Bioanalyzer 2100 analysis of the consecutive fractions from the procedure on top. (**B**) PCA of the data from two analyzed cell populations (vGluT2 and Gad2 neurons, *n* = 3 each) and unbound fraction. (**C**) Heatmap showing relative distribution of TU-Tag TPM values for cell type–enriched mRNAs selected based on data obtained with the original Ribo Tag mouse. Each column represents one biological sample. Z-score for each row was calculated to set the input levels to 0: Z = (x–mean(input))/SD(row), where SD is standard deviation. (**D**) Ribo-Tag assessment of enrichment and/or depletion of 40 genes chosen based on TU-Tag; top 10 genes for each category were selected as follows: enriched in vGluT2 and depleted in Gad2 (LFC_vGluT2_ > 0, LFC_Gad2_ < 0), enriched in Gad2 and depleted in vGluT2 (LFC_vGluT2_ > 0, LFC_Gad2_ < 0), enriched in both Gad2 and vGluT2 (LFC_vGluT2_ > 0, LFC_Gad2_ > 0), depleted in both Gad2 and vGluT2 (LFC_vGluT2_ > 0, LFC_Gad2_ > 0). For each category, the top 10 genes were selected on the basis of the rank metric (|rnk_Gad2_|+|rnk_vGluT2_|); mitochondrially encoded genes were excluded from the analysis. (**E**) Similar to panel D, except that the gene selection was done on the basis of Ribo-Tag and then juxtaposed with TU-Tag. (**F**) Comparison of the overlaps between Ribo-Tag and TU-Tag modalities with respect to the number and direction of significant enrichments (LFC > 0, FDR < 0.1) and depletions (LFC < 0, FDR < 0.1). LFC was calculated by comparing with S1 input supernatants for Ribo-Tag and unbound fractions for TU-Tag affinity purification. All overlaps were deemed highly significant by the hypergeometric test. (**G**) Direct comparison of TU-Tag and Ribo-Tag between two tested neuronal types: Gad2 and vGluT2 cells, showing a high correlation of data, albeit some genes display discrepant behavior between the modalities. (**H**) Scatterplots of log_2_-transformed TPM values from TU-Tag and Ribo-Tag modalities plotted against each other. mtDNA encoded genes were highlighted in blue and labeled with gene symbols. (**I**) Correlation of RNA turnover inferred from Tagger data with SILAC-determined protein turnover times [33] for both tested neuronal subtypes. For each cell subtype, the x-axis represents differences between LFC_Ribo-Tag_ and LFC_TU-Tag_ (both calculated with respect to the corresponding S1 and unbound RNA, respectively), binned into four value ranges: (−∞, −1), (−1, 0), (0, 1), and (1, ∞). The y-axis represents the turnover time inferred from SILAC experiments on primary neuronal cultures [33]. RNAs with faster turnover (as determined by SILAC) tend to have LFC_Ribo-Tag_−LFC_TU-Tag_ lower than RNAs with slower turnover. All differences between groups were significant (Kruskal-Wallis test with post hoc Dunn test, see S2 Table for detailed test results). The raw data on which panels C, D, E, and I are based can be found in S1 Data. CT, control; FDR, false discovery rate; Gad2, glutamic acid decarboxylase 2; LFC, Log_2_ fold change; mtDNA, mitochondrial DNA; MTSEA,methyl thiosulfonate ethylammonium; PCA, principal component analysis; RNAseq, RNA sequencing; SILAC, stable isotope labeling with amino acids in cell culture; TAG, Tagger; TPM, transcripts per million; vGluT2, vesicular glutamate transporter 2.

Despite these challenges, we established a foundation protocol for TU-Tagging in Tagger mice after exploring variations of 4-TU delivery and affinity purification (timing and sequences of steps, ratios, and concentrations of core components, buffers, etc.). We focused on nonoverlapping GABAergic and glutamatergic neuronal subpopulations because we expected a large set of genes to be differentially expressed for cell markers, and because these cell types have different electrophysiological properties, common housekeeping mRNAs may demonstrate different labeling rates between cells. Following a 5-hour labeling period, mice were humanely killed and their brains removed and flash frozen. To facilitate future experiments combining multiple Tagger components, we started with S1 homogenate fractions as prepared for Ribo-Tag and Ago-Tag purifications (Fig 5A). Total RNA was purified from S1 fractions using a combination of organic extractions and silica columns. Purified RNA was then fragmented to reduce signal from sparsely labeled RNA (presumably background labeling), then biotinylated and affinity purified with streptavidin-coated magnetic beads (Fig 5A). Ribosomal RNA constitutes a vast majority of cellular RNA but is not of interest for our purposes, so we established a strategy to remove it using RNaseH digestion [32]. To test for cell type specificity, we performed RNAseq on the affinity purified RNAs and their related unbound counterparts.

PCA analysis revealed clustering of samples according to the cell type of origin (Fig 5B). We observed more variation in the unbound samples, which was later determined to be due to residual contamination with ribosomal RNA not uniformly removed from all samples. However, the affinity-purified samples had no significant ribosomal contamination; hence, they formed tighter clusters. Because the current TU-Tagging method is labor intensive with many possibilities for an accidental deviation from the protocol, the entire process was repeated with a new group of mice. Despite being prepared several months later, the second batch was remarkably similar (S5A Fig). Detailed analysis revealed a batch effect, but the differences in cell type of origin showed a greater contribution to the variation between replicates than did the differences between batches (S5B Fig). We then evaluated the relative enrichments and depletions of mRNAs using the same gene list that was used to validate the Ribo-Tag modality (S1 Table). Remarkably, all mRNAs matched the trends observed in Ribo-Tag (Fig 5C). We then further cross-compared TU-Tag with Ribo-Tag by selecting RNAs based on arbitrarily defined criteria instead of cell type markers. The top 10 RNAs for each of the following four groups for each modality were selected: (1) enriched in vGluT2 and depleted in Gad2, (2) enriched in Gad2 and depleted in vGluT2, (3) enriched in both Gad2 and vGluT2, and (4) depleted in both Gad2 and vGluT2 (see Methods section for details of the selection). RNAs selected within the TU-Tag modality showed similar trends in the Ribo-Tag domain (Fig 5D), and the same was the case for the reverse cross-comparison (Fig 5E). We observed some discrepancies between the two domains, some of which may be due to batch effects, but also because the two labeling methods are expected to capture slightly different pools of mRNAs. Overall, we found significant overlaps across all RNAs that showed (compared with the reference samples) (1) enrichments in both domains (Log_2_ fold change [LFC] > 0 and false discovery rate [FDR] < 0.1, overlap of 53.2% and 41.5% for Gad2 and vGluT2, respectively) and (2) depletions in both domains (LFC > 0 and FDR < 0.1, overlap of 59.4% and 46.6% for Gad2 and vGluT2, respectively) (Fig 5F). Noteworthy, all four overlaps had incalculably low *p*-values when a hypergeometric test was applied. A similar trend was observed when we directly compared both analyzed neuronal types, Gad2 and vGluT2, across both modalities (Fig 5G). In this case, there was a high correlation between the cell types (*R* = 0.89), but genes showing the opposite direction of change between the domains were also detected (Fig 5G). Altogether, after systemic administration of 4-TU, RNAs in specific brain cell types were labeled specifically and sufficiently for affinity purification, setting a foundation for future TU-Tagging experiments in adult mouse brains.

After applying Ribo-Tag data to confirm the robustness of Tagger’s TU-Tag, we wondered if we could gain additional information by discriminating differences between RNAs captured by the two methods. This seemed conceivable, as each of those methods labels and captures RNAs in different ways, and because the target RNAs comprise different fractions of the total cellular pool. For example, a substantial proportion of mRNAs are not associated with intact ribosomes but are instead contained in mitochondria or in granules for sequestration or transport. As a result, such RNAs will likely escape capture by Ribo-Tag. Furthermore, ribosome-bound and ribosome-free transcripts likely differ in synthesis and degradation rates. Finally, the amount of RNA bound to ribosomes may not directly correspond with the overall transcription rate, e.g., for long-lived mRNAs during an acute transcriptional response. In view of the above, Ribo-Tag and TU-Tag should mutually complement each other. We strived to synergize the TU-Tag and Ribo-Tag workflows as much as possible, but essential differences in sample processing (e.g., RNA fragmentation early in the TU-Tag purification procedure) were expected to introduce bias. Therefore, we did not directly compare individual Ribo-Tag and TU-Tag cell type–specific samples but rather calculated differential enrichments within each modality and used the calculated LFC values for comparisons. It is important to mention that, due to technical limitations, we performed poly(A)+ RNA enrichment for the Ribo-Tag materials. Therefore, as histone mRNAs are known to lack poly(A) tails and were underrepresented in our Ribo-Tag samples, we disregarded histone RNAs in the analysis to reduce the potential bias.

Interestingly, a difference between the modalities was especially prominent for a small, yet highly expressed pool of genes encoded in mitochondrial DNA (mtDNA). These genes (encoding components of the electron transport chain) are transcribed and translated in mitochondria. Because Rpl22 and the Ribo-Tag component (Rpl22-HA) of Tagger are absent from mitochondria, mitochondrially encoded mRNAs were barely detected, if at all, in the Ribo-Tag fractions of vGluT2- and Gad2-Tagger brains (Fig 5H).

Because TU-Tag labels RNA following a pulse of exogenous 4-TU, the extent of labeling would depend on several factors, such as RNA turnover rate. Even though we had little information about dynamics of 4-TU metabolism after subcutaneous administration (especially about the time until the peak concentration in the brain would be reached and the expected clearance time), we expected that long-lived and short-lived RNAs might be labeled differently. If the peak concentration was reached quickly and if the clearance was fast, it might be impossible to detect short-lived RNAs after a certain time postinjection (e.g., 5 hours). On the other hand, longer retention of 4-TU (and thus a longer effective labeling window) would favor RNAs with shorter half-lives and result in a higher proportion of such RNAs becoming labeled. To determine which of the above trends is true, we first assumed that the relationship between Ribo-Tag and TU-Tag modalities could be used to infer RNA turnover times. As our metric reflecting the turnover, we have chosen the difference between LFC_Ribo-Tag_ and LFC_TU-Tag_ (both LFCs calculated with respect to the corresponding reference fraction, so S1 and unbound RNA, respectively). Our second assumption was that the turnover of coding RNAs will be, at least generally, a good predictor for inferring the turnover of the corresponding encoded proteins, and vice versa. Because our Ribo-Tag and TU-Tag data were from neuronal subtypes, we compared them with protein turnover rates calculated using stable isotope labeling with amino acids in cell culture (SILAC) in primary neuronal cells, focusing on transcripts encoding proteins that were quantified by SILAC in the study of Heo and colleagues [33]. We then divided mRNAs based on our inferred RNA turnover metric (LFC_Ribo-Tag_ − LFC_TU-Tag_) into four bin ranges: (−∞, −1), (−1, 0), (0, 1), and (1, ∞), and compared mean RNA turnover in each of the bins with the turnover of corresponding proteins determined by SILAC (Fig 5I). RNAs encoding proteins with faster turnover times tended to have LFC_Ribo-Tag_ − LFC_TU-Tag_ differences lower than RNAs encoding proteins with slower turnover. Although the turnover of protein is affected by different mechanisms and would not always reflect the stability and/or half-life of the encoding RNA, we observed a clear overall trend (Fig 5I), and the differences between each group and for each cell type were significant (Kruskal-Wallis test with post hoc Dunn test, see S3 Table for test results). Therefore, in spite of many technical nuances, TU-Tagging is robust and labels overlapping yet different populations of RNAs than Ribo-Tag, thereby greatly complementing the latter methodology.

### Purification of nuclei from specific cell types

We previously applied region- and neuron-specific analysis of chromatin modifications to investigate the consolidation and maintenance of memory [34]. In that study, we employed a universal neuronal marker and could not distinguish between excitatory and inhibitory nuclei, thereby motivating us to include the Nuc-Tag component in Tagger.

To facilitate fluorescence activated cell sorting (FACS) enrichment of cell type–specific nuclei, we incorporated RFP-NLS into Tagger mice. We chose mKate2 for the RFP because it is small (28 kDa) and its native fluorescence is resistant to quenching by formaldehyde fixation [35], thereby providing compatibility with our chip protocols, typically including formaldehyde treatment [34,36]. As endogenous red fluorescence of mKate2 penetrates tissues very well, it is also a useful in vivo imaging tool [37]. We avoided fusing nuclear proteins to RFP to achieve nuclear localization because we found that protein fusions with nuclear matrix targeting signals or nuclear Lamin B1 either performed poorly or disturbed the nuclear structure, and because some fusions, such as with histones, can result in toxicity [18,38]. Thus, we directed RFP to the nucleus by three tandem NLSs only.

**Fig 6:**
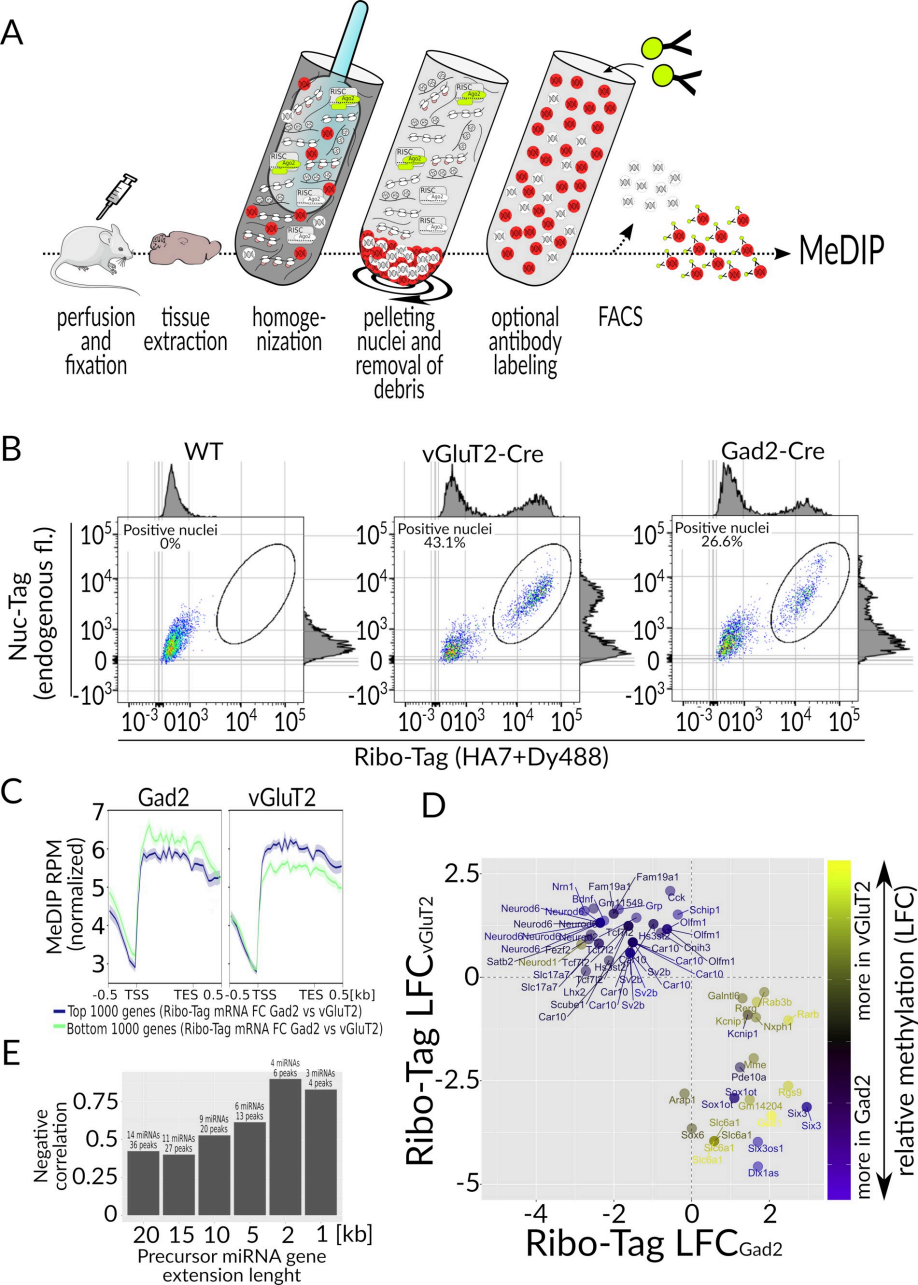
Nuc-Tag. (**A**) Overview of the procedure. Mouse is perfused with 10% formalin and the brain is extracted and homogenized. Nuclei are pelleted to remove the cytoplasmic content. Then, the nuclei are resuspended, optionally labeled with antibodies, and subjected to FACS. From the enriched fraction of nuclei, DNA is isolated and subjected to MeDIP sequencing. (**B**) Representative FACS scatterplots of the fixed nuclei suspensions from WT, vGluT2-Tagger, and Gad2-Tagger brains, assayed for Rpl22-HA (x-axis) and Nuc-Tag endogenous fluorescence (y-axis). (**C**) Aggregate plots of methylation signal of the top 1,000 and bottom 1,000 differentially expressed genes, ordered by *rnk* = LFC*(−log(*FDR*)) (as determined by Ribo-Tag). (**D**) Comparison of Ribo-Tag data and Nuc-Tag (MeDIP) data; genes with |LFC_Gad2 versus vGluT2_| > 2, corresponding FDR < 0.1) and differentially methylated (|LFC_MeDIP_| > 1, FDR_MeDIP_ < 0.1) were plotted; relative methylation differences are reflected by the continuous color scale. (**E**) Correlation of differential methylation and miRNA gene expression, depending on the distance of the methylation peak from the gene TSS. The raw data related to panels D and E can be found in S1 Data. Ago2, Argonaute 2; Cre, causes recombination; FACS, fluorescence activated cell sorting; FDR, false discovery rate; fl, fluorescence; Gad2, glutamic acid decarboxylase 2; LFC, Log_2_ fold change; MeDIP, methylated DNA immunoprecipitation; miRNA, micro RNA; RISC, RNA-induced silencing complex; Rpl22-HA, large subunit ribosome protein 22-hemagglutinin (Ribo-Tag protein); RPM, reads per million; TES, transcription end site; TSS, transcription start site; vGluT2, vesicular glutamate transporter 2; WT, wild-type.

Examination of brain slices revealed that RFP-NLS was restricted to the nucleus (Figs 1D, 2A and 2B). Fluorescence was sufficiently strong for deep in vivo multiphoton imaging of the mouse brain to at least 300 μm (S3A Fig). We then wondered if, in the context of damaged tissue, the transgene would continue to be expressed. Indeed, RFP-NLS fluorescence remained stable in close proximity to laser-lesioned tissue (S1 Movie, S3B, S3C and S3D Fig), indicating Tagger ought to be suitable for studies of diseased and damaged tissues. Despite the stable expression in lesioned tissue, but in line with a previous report [39], RFP-NLS rapidly diffused out of the nucleus upon cell lysis of unfixed tissues. This happened with several compositions of lysis buffer but could be prevented through fixation of the brain by transcardial perfusion with 10% formalin prior to homogenization [39]. Thus, FACS purification of nuclei using the Nuc-Tag element of Tagger requires formaldehyde fixation prior to homogenization. FACS analysis of nuclei from pre-fixed brains revealed a high overlap of HA^+^ and RFP-NLS^+^ populations (Fig 6B). Finally, upon FACS of Tagger^+^ nuclei, the proportion of labeled nuclei matched the numbers we expected based on their relative abundance in the brain (Fig 6B).

We further boosted the separation of Tagger^+^ from Tagger^−^ nuclei by supplementing the fluorescence of RFP in fixed Tagger^+^ nuclei with HA immunofluorescence labeling, because ribosomal subunits are assembled there. The proportions of RFP^+^/HA^+^ nuclei directly correlated with the proportion of targeted cells in the brain (Fig 6B). Regions of methylated DNA were then immunopurified (MeDIP) and sequenced. To confirm that FACS isolated nuclei were indeed derived from the cell types of interest, we first looked at aggregate plots of MeDIP signal on the top 1,000 and bottom 1,000 differentially expressed genes in Gad2 and vGluT2 neurons, as determined by Ribo-Tag. As expected, MeDIP signal was inversely correlated with the differential expression (Fig 6C). Out of genes that were at least 2-fold higher expressed in vGluT2 compared with Gad2 cells (LFC_vGluT2 versus Gad2_ > 1, FDR < 0.05), all were hypermethylated in Gad2 cells as compared with input material, with only one methylation peak in a gene *Neuronal differentiation 1* (*Neurod1*) showing the opposite trend (Fig 6D). Likewise, most genes that were expressed at least 2-fold higher in Gad2 cells compared with input (LFC_Gad2 versus vGluT2_ > 1, FDR < 0.05) were hypermethylated in vGluT2 cells (Fig 6D). We also did a similar comparison using both Ribo-tag and TU-tag data, highlighting differences between the cell types (Gad2 versus vGluT2) instead of comparing first to the input. Similarly, the majority of mRNA-level differences matched the expected methylation pattern (S6 Fig). To complete our evaluation, we also examined correlations of miRNA expression and DNA methylation between Gad2 and vGluT2 neurons. To this end, we identified methylation peaks surrounding miRNA precursor genes and calculated how the mean changes of methylation of these genes correlated with miRNA expression levels. As expected, we observed a negative correlation that increased with decreasing size of the genomic window of interest surrounding the gene (Fig 6E).

As we expect formaldehyde fixation will impair the remaining components, we also developed an alternative approach to purify nuclei from unfixed samples. We accomplished this by immunolabeling Rpl22-HA (S2F Fig) because ribosomal subunits are assembled in the nucleus and are too large to passively diffuse through nuclear pores, even without fixation [40]. Therefore, Tagger enables the capture of nuclei and, thus, extraction and analysis of DNA from specific cell types. Moreover, data can be synergized with the remaining three modalities of the system.

## Discussion

The immense diversity of mammalian cells and their multilayered systems of gene expression regulation pose a huge challenge for research. This challenge is especially notorious in neuroscience, because even a small region of the brain is extremely heterogenous [41]. Many brain functions are poorly understood and most brain diseases are incurable. Alzheimer's, Parkinson’s and most other protein misfolding-associated neurodegenerative diseases are known to affect specific cell types of the brain [42,43] and are widely reported to affect gene expression at multiple levels [44–46]. There is, therefore, a long-standing need for better tools to decipher the relationships between multiple levels of gene expression in specific cell types of complex tissues. In recent years, omics technologies have become a fundamental part of this toolkit. Integration of in vivo data from multiple levels of gene expression would be most informative if they were acquired from the same individual specimen, enabling correlations of intra-individual variations across those levels. While this is feasible for total tissue materials (e.g., mRNA, miRNA, protein, etc.), it becomes increasingly difficult when transgenic technologies are employed for capturing the analytes from specific cell types. Simply combining mouse lines was problematic, as use of Cre/LoxP or similar systems may lead to unwanted chromosomal rearrangements, and transgenes in different chromosomal loci will most likely have different expression patterns. Moreover, many transgenic tools (e.g., miRAP and isolation of nuclei tagged in specific cell types [INTACT]) were targeted to the same genomic location—Rosa26 [47], further limiting the available combinatorial strategies. Due to these difficulties, there is still a need for tools to isolate different types of nucleic acids from specific cells of complex tissues. We sought to address this need by engineering the Tagger knock-in mouse line.

The main advantage of Tagger is the blending of four cutting-edge methodologies—miRAP [11], INTACT [15], RiboTag [10], and TU-Tagging [14]—into one mouse line. A previously reported mouse line carrying a combination of similar molecular tools—NuTRAP—enables the purification of nuclei (Nu) and tagged ribosomes (translating ribosomes affinity purification [TRAP]) [18]. Tagger extends this technology by adding cell type–specific miRNA capture and RNA pulse labeling.

Somewhat surprisingly, the efficiency of Ribo-Tag from Tagger was similar to that of the original RiboTag line. Although Rpl22-HA in Tagger appeared to be a little more abundant, we expected reduced efficiency, because upon activation of RiboTag, a WT Rpl22 allele is removed, reducing competition for Rpl22-HA to integrate into ribosomes. Nonetheless, we found very high yields of RNA capture and specificity that was essentially the same. We also found that Ago-Tag performed similarly well compared with the predecessor miRAP mouse, based on amounts captured and analyzed on the bioanalyzer and the specificity. TU-Tag was more difficult to compare with the predecessor mouse line, as it was used to study very different cell types. Nonetheless, we saw very good specificity and we captured enough RNA for detection with a bioanalyzer, in contrast to the previous line. Compared with its predecessors, INTACT and NuTRAP, Nuc-Tag performed less well as it required fixation to function as a tag to sort nuclei, but it was nevertheless functional.

An important feature of Tagger is its flexibility. Protocols introduced here can easily be modified to accommodate new emerging needs. For example, we successfully purified both Ribo-Tag–and Ago-Tag–associated RNAs from the same aliquot of S1, by first capturing miRNA using anti-FLAG-Ago2 magnetic beads and then using the unbound fraction as input for the Ribo-Tag purification. This increases the overall duration of the preparation by about 2 hours but may be useful to maximize RNA yields of tissue samples that are either small or contain very few target cells. Moreover, each component of Tagger may be regarded as a stand-alone tool. As the performances of Ribo-Tag, Ago-Tag, and TU-Tag modalities are comparable with their predecessors, we see Tagger as a viable solution for investigators who aim to embark on any of these methods, with the option of implementing additional modalities later.

Importantly, by gaining more information from individual mice, Tagger results in a refinement of information obtained from each mouse, potentially leading to a reduction in the total number of animals needed, in accordance with ethical guidelines for the use of animals in research. Such a refinement and reduction will be especially beneficial in studies requiring substantial investments in individual mice, such as experiments involving aging, behavioral training, or tedious surgical procedures.

### Future refinements

Our current work demonstrates that the TU-Tag component of Tagger largely labels RNAs in the cells of interest, while also providing information complementing Ribo-Tag. For example, mitochondrially encoded genes were preferentially captured by TU-Tag (Fig 5H). Because mitochondrial metabolism is compromised in many human diseases [48], including neurodegeneration [49], TU-Tagging may provide a novel tool to measure this impairment in disease models, which would be missed by Ribo-Tag studies.

Applying new methods may further improve the potential/specificity of TU-Tag. The strategy we employed depletes off-target (nonspecifically labeled) RNAs by fragmenting RNA before affinity purification. Off-target RNAs have many fewer thiol groups, whereas on-target (specifically labeled) RNAs have many more thiols. However, a newly developed series of methods based on direct quantification of the number of thiol groups could conceivably be applied to Tagger. These methods are based on a chemical conversion of thiolated nucleotides, similar to that used for bisulfite sequencing of DNA [50,51]. The value of these chemical conversion methods has already been demonstrated in a study of RNA transcription and decay kinetics in cell culture [29]. Application of these methods to Tagger would provide at least three improvements. First, the necessity to fragment RNA would be eliminated, thereby improving yields. Second, the bias we observed comparing total RNA libraries for TU-Tagging, which required fragmentation, with the unfragmented Ribo-Tag samples, would be eliminated. Third, RNA enriched with the biotin-based capture could then be segregated into categories of highly labeled and therefore on target, or lowly labeled and therefore off target and filtered out. Any of these would be a great improvement and would enhance studies of tissue samples that are small or carry few cells of interest. Moreover, the capacity to employ multiple components of the Tagger could be exploited further. For example, mRNA could first be captured by Ribo-Tag, and then a portion of these mRNAs could be analyzed with a chemical conversion method to determine which were newly synthesized and potentially determining cell type–specific kinetics and turnover rates of individual transcripts.

Nuc-Tag is the Tagger component most different from predecessor methods. In both NuTRAP and INACT, nuclear envelope fusion proteins label cell type–specific nuclei, which can be purified with affinity purification or FACS [15,18]. To mitigate risks of expression and gene-targeting problems, Tagger uses an unanchored RFP fused with a triple NLS as a necessary trade-off because the coding sequence for the small (28 kDa) protein better fit into an already large knock-in construct (S1A Fig). Although Nuc-Tag requires formaldehyde fixation, the RFP endogenous fluorescence is highly resistant to cross-linking, preserving sufficient signal for FACS and histology. This design also reduces the risk of interference with nuclear functions, as there is no endogenous protein that might compete with the fusion tag for binding partners or subnuclear localization. Although the necessity of fixation may be seen as a downside, it is partially compensated for by its excellent performance in deep in vivo two-photon imaging. Furthermore, fixation may prove beneficial to retain other molecules of potential interest (e.g., nuclear RNA or proteins). Alternatively, it is also possible to isolate unfixed cell type–specific nuclei with Tagger. This can be accomplished through HA labeling, as Rpl22-HA (Ribo-Tag) is shuttled to the nucleus, where ribosomal subunits are assembled. The assembled ribosome complexes are too large to passively diffuse out, and thus Rpl22-HA might be used as an alternative nuclear tag for the capture of native nuclei (S2F Fig) [40]. In this setup, it would be possible to purify native nuclei and, in parallel, co-capture Ago-Tag, Ribo-Tag, and TU-Tag modalities from the same sample.

We also anticipate the combined use of Cre and Flp drivers for the incorporation of intersectional labeling of target cells to be an important refinement. In many cases, this will result in a very small number of cells being labeled. Although we have not tried to use such sparsely labeled Tagger samples, we can envision there being new challenges. However, these challenges should be easily met by dissecting tissues to remove areas lacking positive cells, and possibly combining such dissected tissues from multiple mice.

### Tagger in the context of other methods

A separate group of methods may be viewed as competing with technologies like Tagger. These methods investigate gene expression in thousands of individual single cells in parallel, the most notable of which is single cell RNAseq (scRNAseq). An important advantage of scRNAseq is its applicability to WT samples, including human tissues. Tagger requires expression of foreign DNA, which is most stably provided by transgenesis and, in its current form, requires at least one recombinase, currently provided by an additional transgene. Another advantage of scRNAseq is that there is no cellular heterogeneity to obscure information, as is the case in Tagger, for which specific populations will necessarily carry some inherent heterogeneity.

However, scRNAseq technologies are not without their downsides. With scRNAseq, the types of nucleic acids studied are heterogenous and typically limited. As a result, information such as whether detected mRNAs are mature or attached to ribosomes remains obscured. Similarly, single cell methods for miRNAs and chromatin are seldom studied, mainly for technical reasons. Another limitation of scRNAseq is the comparatively low coverage of the transcriptome, which results in moderately and lowly expressed genes being undetected [52]. Moreover, dissociation of samples needed to obtain single cell suspensions comes at the expense of biases due to material losses (e.g., dendritic or axonal RNAs) and artifactual changes in gene expression induced by the dissociation procedure. In this context, a major advantage of Tagger is the instant homogenization of tissue upon dissection or flash freezing of the specimens for later analysis. Finally, pulse labeling methods such as TU-Tagging would be difficult to implement for in vivo scRNAseq experiments, because the majority of RNA would be unlabeled and a biochemical enrichment strategy would be needed. Tagger preserves the native states of the analytes by circumventing the inherent dissociation step in scRNAseq methods.

While many of the scRNAseq limitations are acceptable in studies aimed at elucidating a tissue’s cellular diversity and taxonomy [41], they become problematic when it comes to analyzing functional relationships between cells, in which shifts in expression of moderate and lowly expressed genes often play key roles [53]. Therefore, we view Tagger and scRNAseq methods as being complementary. In one case, a good separation of cell types can be obtained at the expense of mixing of nucleic acid types, whereas in the other case, a good separation of nucleic acid types can be obtained at the expense of the mixing of related cell types.

In spite of its limitations, Tagger offers unprecedented versatility, with potential for further development. The modalities of Tagger can be used to extract analytes related to distinct levels of gene expression. These four components, akin to tools in a Swiss army knife, can be combined as seen fit for individually customized, cell type–specific multiomics experiments. In this way, the Tagger expands the amount, quality, and completeness of information attained from every invaluable mouse.

Gene expression data presented in this report can be visualized using a shiny app accessible at https://shiny.it.liu.se/shiny/TaggerApp. Raw next generation sequencing (NGS) data are available from NCBI GEO (GSE123422).

## Materials and methods

### Ethical statement

Ethical permissions for this work were granted by the Landesamt für Natur, Umwelt und Verbraucherschutz Nordrhein-Westfalen, 84–02.04.2012.A192, 84–02.04.2017.A016, 84–02.04.2013.A128, 84–02.04.2017.A098, and 84–02.04.2013.A169. All experimental procedures were performed in accordance with the internal regulations of the DZNE.

### Tagger mouse generation

We expected that the use of a recombinase system in a context of multiple transgenes would lead to intergenic rearrangements and/or discrepancies in expression patterns of expressed proteins [8]. These unpredictable and hard to control confounders enforced an alternative approach—2A peptide [19], also used successfully in the NuTRAP mouse [18]. In contrast to internal ribosome entry sites (IRESs), which typically result in decreased expression of downstream IRES-driven components [54], which we feared would multiply when using multiple IRESs, 2A peptides efficiently separate multiple proteins with similar stoichiometry and fully retained functions [19]. We designed a transgene comprising HA-tagged ribosomal protein L22 (Rpl22-HA, for Ribo-Tag), Uracil phosphoribosyltransferase from *T*. *gondii* (TgUPRT, for TU-Tag), red fluorescent protein with a triple NLS (RFP-NLS, for Nuc-Tag), and FLAG-V5-tagged Argonaute2 (FLAG-V5-Ago2, for Ago-Tag). This cassette was placed under the control of the cytomegalovirus:chicken actin fusion promoter (CAG) promoter and two transcription-terminating STOP cassettes—Cre-dependent (LSL) and Flp-dependent (FNF). In the Tagger transgene, protein coding cistrons were separated by sequence encoding the Porcine Teschovirus 2A peptide (P2A) and flexible linkers (GSGSG) for improved separation (Fig 1A and S1A Fig). Because all but the terminal proline of the 2A peptide is retained on the C terminus of upstream proteins, and because the C terminus of Ago2 is critical for its function [55], the construct was designed to avoid a residual 2A peptide on FLAG-V5-Ago2 by placing it in the last cistron. We had less concern for the remaining three components because C-terminal fusions are functional [14,37,56]. We targeted the transgene to the Rosa26 locus, including a ubiquitous CAG driver [57] followed by Flp- (FNF) and Cre-dependent (LSL) terminators (STOP cassettes). The inclusion of two terminators enables transgene activation at the intersection of cell populations expressing Cre and Flp driven by distinct promoters, to precisely target cells based on combinations of cell identity markers or activity [22]. R26LK–thymidine kinase (TK)-Tagger targeting vector and Tagger ORF were generated using molecular cloning and gene synthesis. In the process, a fragment from Ai3 vector (a gift from Hongkui Zeng, plasmid #22797, Addgene, Cambridge, MA), FNF cassette from pFNF vector (a gift from Robert Benezra, plasmid #22687, Addgene, Cambridge, MA), and Ago2 sequence (a gift from Thomas Tuschl, plasmid #10822, Addgene, Cambridge, MA) were used. For cloning of the homology arms, first a fragment of Rosa26 locus was PCR amplified using genomic DNA of the 129S4 strain as a template and AGCGTGGTGGAGCCGTTCTG and AATGTGAATACACTTGTGGTC (R26 homology F and R, respectively) primers. The obtained PCR product was cloned into pSC-A T/A vector (Agilent, Santa Clara, CA) and served as a template for PCR amplification of respective homology arms, creating restriction overhangs. The homology junction was positioned 3 nt upstream of the PAM sequence for the Cas9 sgRNA that subsequently was used for gene targeting. A codon-optimized TK negative selection cassette was synthesized (IDT, Coralville, IA) and inserted to generate the R26LK-TK (HTV) targeting vector. The CMV-Tagger vector (Tagger ORF driven by CMV promoter) is available from Addgene (plasmid 129396).

### Generation of Tagger knock-in mice and mouse breeding

Tagger expression cassette (in R26LK-TK-Tagger vector, details in S1A Fig) was targeted into the Rosa26 safe harbor locus in V6.5 ES cells [58] using CRIPSR/Cas9, as described previously [59]. In brief, 40 μg of HTV linearized with AscI was co-electroporated with 5 μg CRISPR/Cas9 nickase plasmid pX335 (a gift from Feng Zhang, plasmid #42335, Addgene, Cambridge, Massachusetts) encoding sgRNA targeting the homology junction. Correct genomic insertion was verified by PCR across both homology regions (S1B Fig) following PCR screening for the absence of pBlueScript vector backbone to confirm omega-type recombination, and only a single insertion was present (S1C Fig). All PCR screens were repeated on genomic DNA of the mice (S1B Fig). The targeted ES cells were injected in C57Bl/6NCrl (B6 hereafter) blastocysts and transferred to pseudopregnant Crl:CD1 foster mothers. Male chimeric offspring were first bred to B6 females. One chimera was determined to transmit the ES genome to all progeny and was therefore subsequently bred to 129S4 (S4 hereafter) mice to establish the Tagger system in a second genetic background. S4 was chosen because the ES cells are a hybrid of B6 and S4, and it is our lab’s primary strain. Mice carrying both FNF and LSL cassettes (FL-Tagger) were bred to Actb-Flpe mice on a B6 background [60] or to ROSA26-Flpo mice on the S4 background (line #007844, Jackson Labs, Bar Harbor, ME) [61]) to remove the FNF cassettes, creating LSL-Tagger lines. All lines were back-crossed into S4 and B6 backgrounds for a total of seven generations. Genome-wide analysis of 569 SNPs, 355 of which discriminate between B6 and S4, indicated the final backgrounds: B6-FL-Tagger or B6.129S4-*Gt(ROSA)26Sor*^*tm1Jaws*^ (98.6%), B6-LSL-Tagger or B6.129S4-*Gt(ROSA)26Sor*^*tm1*.*1Jaws*^ (97.5%), S4-FL-Tagger or 129S4.B6-*Gt(ROSA)26Sor*^*tm1Jaws*^ (95.8%), and S4-LSL-Tagger or 129S4.B6-*Gt(ROSA)26Sor*^*tm1*.*1Jaws*^ (97.8%). An SNP analysis at generation 3 revealed that the Tagger allele is embedded on the B6 chromosome 6. PGK-Cre [62], PV-Cre [26], Gad2-Cre [25], and vGluT2-Ires-Cre [24] mouse lines were used to activate LSL-Tagger mice, the last 3 congenic for S4. Sequencing data were acquired from Cre-Tagger mice on a mostly S4 background. The Tagger line with no stop cassettes (omni-Tagger or B6.129S4-*Gt(ROSA)26Sor*^*tm1(Tag)*.*2Jaws*^) was crossed seven generations to B6 but not SNP analyzed. Efforts are underway to deposit the mice in a repository. Otherwise, please email WSJ with the subject heading “Acq-Req-Tagger,” and the contents of the message should indicate the specific version of Tagger requested. As part of the material transfer agreement, Evrogen, the intellectual property owner of the mKate2 sequence, requires recipients of Tagger materials to make an additional purchase of an Evrogen-owned plasmid containing mKate2 sequence. This thwarted efforts to deposit the mouse line prior to publication of this manuscript. The authors have no financial or other connections with Evrogen.

#### Immunoblotting

Brain lysates were mixed with 4× lithium dodecyl sulfate (LDS) sample buffer containing 40 mM DTT (lysates were diluted 1:3, and IP magnetic beads were resuspended in diluted sample buffer to obtain 1× final concentration) and denatured at 70°C for 10 minutes prior to loading on 10% or 4%–12% NuPAGE Novex midi gels (Thermo Fisher, Waltham, MA). Gels were run using MES [2-(N-morpholino)ethane sulfonic acid] running buffer at 160 V (110 V for the first 10 minutes) and then were electro-transferred to nitrocellulose membrane (Bio-Rad, Hercules, CA) and submerged in transfer buffer (20% methanol, 25 mM Tris-Cl, 0.19 M glycine) using a Criterion transfer tank (BioRad, Hercules, CA), at 0.7 A for 70 minutes. Membranes were blocked (20–30 minutes at room temperature [RT]) in 5% powdered milk in PBS-T (PBS with 0.05% Tween-20) and then incubated with primary antibody diluted in blocking buffer overnight (O/N) at 4°C. Next, blots were washed 4× with PBS-T and incubated with secondary antibody for 30–60 minutes at RT, followed by 5× PBS-T washes and imaging with the Li-Cor Odyssey imaging system (Li-Cor, Lincoln, NE). For re-probing, membranes were stripped for 10 minutes with Re-Blot mild or strong solution (EMD-Millipore, Burlington, MA), followed by extensive washing and re-blocking. Primary antibodies were as follows: rabbit anti-Dicer1 (dil. 1:1,000, Sigma Aldrich, St. Louis, MO), rabbit anti-HA (H6980, dil. 1:1,000, Sigma Aldrich, St. Louis, MO), goat anti-V5 (ab95038, dil. 1:2,000, Abcam, Cambridge, United Kingdom), mouse anti-S6 (#2317, dil. 1:1,000, CST, Leiden, the Netherlands), mouse anti-Ago2 (H00027161-M01, dil. 1:1,000, Novus Biologicals, Centennial, CO), and rabbit anti-β-actin (A2228, dil. 1:10,000, Sigma, St. Louis, MO). Secondary antibodies were as follows: donkey anti-rabbit IRDye 680RW (1:10,000, Li-Cor, Lincoln, NE), donkey anti-mouse IRDye 800CW (dil. 1:20,000, Li-Cor, Lincoln, NE), and donkey anti-goat IRDye 800CW (1:20,000, Li-Cor, Lincoln, NE).

### Immunofluorescence

Mice were humanely killed with CO_2_ and then transcardially perfused with 10% formalin, and the brains were postfixed for 2 days at 4°C with gentle shaking. Forty-micrometer slices were cut on a cryotome (Leica, Wetzlar, Germany) and stored in cryoprotectant in the freezer until needed. Slices were washed in PBS, permeabilized for 1 hour at RT in blocking buffer (PBS with 5% normal goat serum and 0.25% Triton X-100), and then incubated with primary antibody solution in probing buffer (approximately the same as blocking buffer but containing 10× less Triton X-100) O/N at 4°C with gentle agitation. Then, sections were washed 3 × 10 minutes with PBS (DAPI was added during the second wash step to the final concentration of 0.1 μg/mL). After the last wash step, secondary antibody diluted in PBS with 5% normal goat serum was added for an additional 1 hour of incubation. Finally, slices were washed (3 × 10 minutes) with PBS, each time with gentle agitation, and mounted using Vector hardset mounting medium (Vector Laboratories, Burlingame, CA). For Gad67 staining, Triton X-100 was not used at any step, and the primary antibody incubation was extended to 2–3 days and performed at RT. Slices were imaged on the confocal microscope LSM 700 (Zeiss, Oberkochen, Germany) using a 63× oil immersion objective. For images selected for publication, brightness and contrast were uniformly enhanced using ImageJ. Primary antibodies were as follows: HA-tag, 1:200 (3F10, Roche, Basel, Switzerland), parvalbumin, 1:200 (NB-120-11427, Novus Biologicals, Centennial, CO); Satb2, 1:200 (ab92446, Abcam, Cambridge, UK); and Gad67, 1:800 (MAB5406, Millipore, Burlington, MA). Secondary antibodies (each used at 1:1,000) were as follows: FITC goat anti-rabbit (FI-1000, Vector Laboratories, Burlingame, CA), DyLight-649 goat anti-rabbit (DI-1649, Vector Laboratories, Burlingame, CA), and DyLight-488 goat anti-mouse (DI-2488, Vector Laboratories, Burlingame, CA).

### Immunohistochemistry

Mice were humanely killed with CO_2_ and brains or retinas were carefully removed and immersion fixed with 10% formalin. The brains were postfixed for 2 to 3 days at 4°C with gentle shaking. Brains were then embedded in paraffin, with each cassette containing both controls and transgenic samples of interest to ensure identical staining. Cassettes were cut into 4-μm-thick sections. Sections were dewaxed in xylene and rehydrated in graded dilutions of ethanol (each 5 minutes). Epitope retrieval was performed with a steamer in 0.01 M citrate buffer. Endogenous peroxidase was inactivated with H2O2 treatment (0.3%, 20 minutes). Primary antibodies made in mice were detected with the Mouse on Mouse Elite Peroxidase Kit and NovaRED or VIP substrates (PK-2200, SK-4800, and SK-4600, respectively, Vector Laboratories, Burlingame, CA), and primaries made in Rabbit were detected with the Vectstain ABC-AP kit and Vector Black substrate (AK-5001 and SK-5200, respectively, Vector Laboratories, Burlingame, CA), all used according to the manufacturer’s instructions. Double stains were performed by processing the Rabbit antibody first, blocking free biotin groups with a blocking kit (SP-2002, Vector Laboratories, Burlingame, CA), then proceeding with the mouse antibody. Pictures of sections were made with an AxioCam camera mounted onto a AXIO Observer A1 microscope with Zen 2012 software (Zeiss, Oberkochen, Germany). Primary antibodies were as follows: rabbit RFP, 1:500 (R10367, Thermo Fisher, Waltham, MA); mouse V5-tag, 1:200 (R960-25); and mouse HA-tag, 1:2,000 (HA7, Sigma, St Louis, MO).

### Co-capturing of mRNA and mature miRNA

We previously modified our Ribo-Tag protocol to increase its specificity [63]. The procedure involves preparation of 10% brain homogenate in an isotonic lysis buffer with detergent, removing nuclei and cell debris by centrifugation to generate a supernatant fraction (hereafter S1), then IP of intact ribosomes together with bound mRNA [10] (Fig 3A). Deep-frozen brain tissue was used to prepare 10% (w/w) homogenate in PSB containing 50 mM Tris (pH 7.5), 100 mM KCl, 12 mM MgCl_2_ and 1% Nonidet P-40, 1 mM DTT, 100 U/mL Ribolock RNase inhibitor, 100 μg/mL cycloheximide, and 1 tab/5 mL SigmaFast protease inhibitor cocktail (with EDTA). One hemisphere was used per replicate, and homogenates were prepared in Potter-Elvehjem homogenizers using a motorized (450 rpm) pestle and then cleared by centrifugation (10,000*g*/10 minutes/4°C) to remove nuclei and cell debris and obtain the S1 supernatant. After removing 50 μL of sample for input RNA isolation, S1 was pre-cleared with Protein G Dynabeads (PGDBs; Thermo Fisher, Waltham, MA) for 30 minutes at 4°C on a rotator and 300 μL of S1 was incubated with 5 μg of HA 12CA5 mAb (Roche, Basel, Switzerland) at 4°C for 45–60 minutes on a rotator (Ribo-Tag sample), and the remainder of S1 was mixed with PSB-equilibrated M2 Magnetic beads (Sigma, St. Louis, MO) and left for a 150-minute incubation in the same conditions (Ago-Tag sample). The Ribo-Tag sample was then transferred to a prepared equivalent of 37.5 μL total bead suspension PGDB (PSB-equilibrated) and incubated as above for an additional 100 minutes. Afterwards, both Ribo-Tag and Ago-Tag beads were washed 3 × 5 minutes in High Salt Buffer (HSB) containing 50 mM Tris (pH 7.5), 300 mM KCl, 12 mM MgCl_2_ and 1% Nonidet P-40, 1 mM DTT, 50 U/mL Ribolock RNase inhibitor (Thermo Fisher, Waltham, MA), 100 μg/mL cycloheximide, and 1 tab/20 mL, and an additional 3 × 5 minutes in Extra High Salt Buffer (EHSB; identical to HSB but containing additional 300 mM NaCl). We found that increasing ionic strength, and thereby the stringency, of the wash buffer did not reduce the yields (S4B Fig). During each wash, beads were rotated gently at 4°C. Following removal of the last wash solution, 700 μL Qiazol (Qiagen, Hilden, Germany) was added and the beads were incubated for 15 minutes at RT with vigorous (>1,000 rpm) agitation. RNA was extracted from Ago-Tag, Ribo-Tag, and input samples using miRNeasy Micro kit (Qiagen, Hilden, Germany) and eluted with 14 μL (Ago-Tag) and 28 μL water (Ribo-Tag and input samples).

### TU-tagging

A 1 M solution of 4-TU (Sigma, St. Louis, MO) in DMSO was stored in small aliquots at −20°C. Within 30 minutes of subcutaneous injection, 4-TU solution was diluted 1:20 into 50 mM HEPES, pH 8.8, prewarmed to 45°C. Therefore, the working solution was maintained at approximately 45°C until it was loaded into a 1-mL syringe with a 25-gauge needle immediately prior to injection of 0.5 mL. Mice were humanely killed 5 hours later via CO_2_ asphyxiation and brains were rapidly removed and frozen on dry ice. TU-Tagged RNA purification started with S1 produced as done for Ribo-Tag samples. RNA was extracted with a phenol:choloroform method, followed by purification with RNeasy mini columns (Qiagen, Hilden, Germany). Approximately 100 μg of total RNA was fragmented with the fragmentation kit (NEB, Ipswich, MA) and cleaned with the RNAeasy minelute kit (Qiagen, Hilden, Germany). Fragmented RNA was biotinylated as done in [64] using MTSEA-biotin-XX (Biotium, Fremont, CA), and then free biotin was removed with two washes of chloroform and isopropanol precipitation. Biotinylated RNA was affinity purified with streptavidin-linked magnetic Dynabeads C1 (Thermo Fisher, Waltham, MA) in the manufacturer’s recommended buffers. Affinity-purified RNA was eluted in binding and wash buffer supplemented with 1% 2-mercaptoethanol and purified with RNeasy micro columns (Qiagen, Hilden, Germany). Unbound RNA was purified by isopropanol precipitation. Ribosomal RNA was removed from affinity-purified and unbound fractions using an RNAse H method described before [32].

### Nuclei isolation

Mice were anesthetized and transcardially perfused with 4% PFA [39]. Brains were extracted from skulls just prior to homogenization, which started 9 minutes post-perfusion. Except fixation time, all steps were done on ice or at 4°C. Hemispheres were separated, placed on a Petri dish filled with PBS, cut into 4–5 pieces with a scalpel, and transferred into a Dounce homogenizer filled with homogenization buffer (2–2.5 mL per hemisphere) containing 120 mM Tris (pH 7.5), 50 mM sucrose, 20 mM KCl, 5 mM MgCl_2_, 50 mM NH_4_Cl, 0.05% Nonidet P40, and 1 mM DTT. Tissue was homogenized by approximately 20 strokes with pestle A (loose) and then between 40 and 50 strokes (slowly, not to damage nuclei) with pestle B (tight). Homogenate was transferred into a 50-mL falcon tube through a 100-μm strainer, washed with an additional 1 mL of homogenization buffer, and then distributed into 1.5-mL tubes for centrifugation (400*g*/5 minutes). Pellets containing nuclei were resuspended in wash buffer (1× DPBS [with Ca^2+^/Mg^2+^], 10 mM Tris [pH 7.5], 5 mM MgCl_2_, 1 mM DTT) and distributed further into 1.5-mL tubes for antibody incubation in a wash buffer supplemented with 1% BSA and 3% normal goat serum (the same antibodies were used as in the case of the native procedure). After 2 hours of incubation, nuclei were washed 3× with the same buffer, but without BSA and normal goat serum. During each wash, nuclei were very carefully resuspended by gentle pipetting and then centrifuged (400*g*/4 minutes). When using unconjugated primary antibodies, nuclei were washed again 3× and incubated for 30 minutes with secondary antibody diluted in the same buffer. During the last incubation, samples were labeled with DAPI (10–20 ng/mL) for the last 10 minutes, washed 2–3× and filtered through a 70-μm strainer prior to FACS. If using only native fluorescence, antibody incubation steps were skipped and the nuclei were stained with DAPI, washed 2× with homogenization buffer containing 250 mM sucrose, and then resuspended for FACS in wash buffer supplemented with 1% BSA.

### Sequencing

#### MeDIP sequencing

DNA was isolated from FACS-sorted cell type–specific (Nuc-Tag+) nuclei using the shearing method with a Bioruptor plus sonication device (cat. B01020001, Diagenode, Denville, NJ) and purified using SureClean plus (cat. BIO-37048, Bioline,) as described previously [34]. Samples containing 100 ng of DNA were end-repaired, ligated with adapters (NEBNext Ultra II DNA Library Prep Kit for Illumina, cat. E7645, NEB, Ipswich, MA), and cleaned up using Agencourt AMPure XP beads (cat. A63881, Beckman Coulter, Brea, CA). A total of 2 μL of each adapter ligated sample input (diluted 1:100) was used to estimate the adapter ligation quality by qPCR. In brief, samples were mixed with index (#E7350, NEB, Ipswich, MA), universal primers, SyBr dye, and 2X NEBNext Ultra II Q5 master mix containing high fidelity DNA Polymerase (cat. M0544S, NEB, Ipswich, MA), and analyzed by qPCR. End-repaired and adapter ligated DNA was used for the MeDIP reaction using a Methylated DNA IP kit (cat. D5101, Zymo Research, Irvine, CA) according to the manufacturer’s protocol. Methylated DNA IPed samples were further amplified to prepare libraries for sequencing. In brief, mid-log ct values were calculated for IPed samples using qPCR to determine the number of amplification cycles. Samples were mixed with the Q5 NEB Next master mix, Index and Universal primer, and amplified for mid-log ct—2 PCR cycles. The libraries were purified by SureClean (Bioline, London, UK) precipitation, resuspended in 10 mM Tris (pH 8), and library sizes were checked using a bioanalyzer 2100 (Agilent, Santa Clara, CA). Libraries were also checked for MeDIP enrichment by qPCR as described in [34]. Final libraries were sequenced in Hiseq-2000 (Illumina, San Diego, CA) according to the manufacturer’s protocol.

#### Ago-Tag RNAseq

For Ago-Tag sequencing, libraries were generated using TruSeq small RNA library preparation kit (Illumina, San Diego, CA) following the manufacturer’s protocol. In brief, 3′ and 5′ adapters were ligated on 1 μg total RNA. Reverse transcription of 6 μL adapter-ligated RNA was carried out using 1 μL RNA RT primer, 2 μL 5× first strand buffer, 0.5 μL of 12.5 nM dNTP mix, 1 μL 100 mM DTT, 1 μL RNase inhibitor, and 1 μL SuperScript II reverse transcriptase. DNA was amplified using PCR, and index primer was added. Samples were pooled together, and cDNA construct was purified on 6% PAGE gel. After washing with 70% ethanol, libraries were resuspended in 10 μL of 10 mM Tris-HCl (pH 8.5). Libraries were validated using a High sensitivity DNA bioanalyzer (Agilent, Santa Clara, CA) and sequenced in Hiseq-2000 (Illumina, San Diego, CA).

#### Ribo-Tag sequencing

For Ribo-Tag sequencing, libraries were prepared using TruSeq RNA Library Prep Kit v2 (RS-122-2001 and RS-122-2002, Illumina, San Diego, CA) following the manufacturer protocol. Libraries were validated using Qubit 2.0 Fluorometer (Thermo Fisher, Waltham, MA) and Bioanalyzer 2100 (Agilent, Santa Clara, CA). Quality-checked libraries were sequenced in HiSeq2000 (Illumina, San Diego, CA).

#### TU-Tag RNAseq

RNA-seq libraries of 4-TU labeled samples were prepared using the ScriptSeq V2 RNA-Seq Library Preparation Kit (cat. SSV21124, Illumina, San Diego, CA) following the manufacturer’s protocol. In brief, cDNA was synthesized using ScriptSeq cDNA synthesis primer, StarScript AMV reverse transcriptase, and 100 mM DTT. The 3′ end was tagged using Terminal tagging premix and DNA polymerase. cDNA was purified using 1.8× AMPure XP, and libraries were PCR amplified by adding Illumina adapter sequence and an index in the library. Final libraries were again purified with 1× Agencourt AMPure XP (cat. A63881, Beckman Coulter, Brea, CA) and libraries were validated using Qubit 2.0 (Thermo Fisher, Waltham, MA) and High sensitivity DNA bioanalyzer (Agilent, Santa Clara, CA). Libraries were diluted to 2 nM for sequencing into HiSeq-2500 (Illumina, San Diego, CA).

### Data analysis

For RNAseq and TU-tag RNAseq, reads were quality assessed using the FASTQC (v0.10.1) and aligned to the mouse reference genome (mm10) with Bowtie2 (v2.0.2) using RSEM (v1.2.29) with default parameters. First, the mouse reference genome was indexed using the Ensembl annotations (v84) with rsem-prepare-reference from RSEM software. Next, rsem-calculate-expression was used to align the reads and quantify the gene and isoform abundance. The output of rsem-calculate-expression gives separately the read count and transcripts per million (TPM) value for each gene and isoform. MeDIP-seq data were analyzed as described previously [34]. Briefly, reads were aligned to the mouse reference genome (mm10) using Bowtie (v2.0.2), with default parameters allowing for 2 mismatches using seed alignment. Subsequently, aligned reads were filtered for those that are high quality and either uniquely or multi-mapped (MAPQ ! = [0, 2, 3, 4]). For the analysis of differentially methylated regions (DMRs), the R package MEDIPS (v1.16.0) was used with BSgenome = BSgenome.Mmusculus.UCSC.mm10, extend = 250, shift = 0, uniq = TRUE, window_size = 700, and minRowSum = 35. Methylation was considered significant at FDR < 0.1 using the Benjamini–Hochberg correction for multiple testing and |LFC| > 0.5. sRNA-seq data were analyzed using Oasis 2 [28]. In brief, the FASTQC tool was used for the assessment of raw reads quality. After adapter trimming (TGGAATTCTCGGGTGCCAAGG), reads were filtered for a minimum length of 15 and maximum length of 32 bp. Filtered reads were first aligned to mm10 transcripts, including known miRNAs, novel miRNAs, piRNAs, snoRNAs, snRNAs, rRNAs, and their families using STAR aligner, with no mismatches for reads of length 15–19 nucleotides and allowing for 1 mismatch for the reads of length 20–32 nucleotides. Expression (counts) of these sRNAs served as the basis for differential expression analysis with DESeq2 package (v1.16.1) using the Oasis 2 pipeline. Preprocessed data can be found in S1 Data.

### Differential expression, statistical analysis, and data visualization

Differential expression analysis was carried out using gene read counts with DESeq2 package (v1.16.1) to produce LFC values and corresponding *p*-values (FDR) applying a Benjamini–Hochberg correction for multiple testing [65]. The ranking metric for enrichment or depletion of mRNA was calculated as follows: *rnk* = LFC*(−log(*FDR*)). Significance of gene list overlaps (Venn diagrams in Figs 3F and 4F) was determined with a hypergeometric test (*phyper* R function). Significance of the correlation of Tagger data with SILAC protein turnover times from [33] (Fig 5I) was performed using the Kruskal-Wallis test with post hoc Dunn test (please refer to S2 Table). Heatmaps were made using TPM values or, in the case of small RNAseq, CPM values using R pheatmap package. PCA analysis was performed using variance stabilizing transformation (VST)-transformed normalized count data (calculated using DESeq2 package [v1.16.1]) and visualized using ggplot2 (v3.1.0). Flow cytometry data were visualized using FlowJo (v10.4.2). Bioanalyzer plots (Figs 3B, 4B and 5A) were made using Bioanalyzer 2100 Expert Software. Metagene plots were made using deepTools2 web server [66].

### Multiphoton imaging

Cranial window surgery was conducted under anesthesia (ketamine/xylazine 0.13/0.01 mg/kg body weight, i.p.) with a perioperative dose of an analgesic (buprenorphine hydrochloride 0.05 mg/kg body weight, s.c.). Additionally, mice received an immunosuppressive drug (dexamethasone 0.2 mg/kg, s.c.) to reduce swelling of the brain during the surgical procedure. Eyes were covered with ointment (Bepanthen, Beyer, Leverkusen, Germany) to prevent drying. Mice were head-fixed (MA-6N, Narishige, Tokyo, Japan), and a sufficiently deep anesthesia was assured by testing the paw pinch withdrawal reflex. A heating pad was used to maintain body temperature during the surgical procedure. The head was wiped with 70% ethanol using sterile cotton swabs. Removing a triangular piece of the skin by surgical scissors exposed the skull. The periosteum was carefully removed with a scalpel and the underlying bone was dried thoroughly before it was sealed by applying a layer of dental adhesive (OptiBond FL bottle kit, Kerr, Rastatt, Germany). A circular piece of the bone above the right somatosensory cortex was removed by first marking the position by using a biopsy punch (Ø 4 mm, pfm medical), and, second, carefully drilling along the marked line with a dental drill equipped with a rounded drill head (H71.104.004, Gebr. Basseler, Lemgo, Germany). After rinsing the brain surface with sterile PBS, the bone was replaced by a circular cover glass (DR3, thickness #1, Engelbrecht, Edermünde, Germany), which was adhered to the bone by dental acrylic. A custom-made fixation adaptor was attached to the skull in a distance to the window to allow for fixation of the mouse during imaging. The remaining bone was covered with light-curable flowable composite (GRADIA DIRECT flo, GC, Leuven, Belgium). Imaging was carried out directly after the cranial window surgery under a prolonged anesthesia using isoflurane (1%, Actavis, New Jersey, USA). Mice were head-fixed to a custom-made frame and eyes were covered with ointment. A heating pad was used to maintain body temperature during the imaging session. Images were acquired at a TrimScope II setup (La Vision Biotech, Bielefeld, Germany) equipped with a Coherent Cameleon Ultra II two-photon laser (Coherent, Dieburg, Germany) and a 16× water immersion objective with a numerical aperture of 0.8 (Nikon, Tokio, Japan). Image acquisition was performed with ImspectorPro (La Vision Biotech, Bielefeld, Germany). mKate2 was excited at 1100 nm. Fluorescence emission was filtered (BP575-610) and detected by non-descanned detectors. Before and after the lesion, z-stacks were acquired, starting at the surface of the cortex (300 μm depth; 3 μm z-spacing; x,y resolution of 0.38 μm/pixel). A laser lesion (Ø approximately 50 μm) was set in a depth of 180 μm by focusing the full-powered laser beam on one pixel lasting for 2 seconds. After the imaging session, mice were injected with a lethal dose of pentobarbital (200 mg/kg body weight, i.p.) and perfused transcardially with saline solution followed by 4% (w/v) paraformaldehyde in 0.1 M phosphate buffer. Brains were removed and postfixed in 4% paraformaldehyde for 24 hours.

## Supporting information

S1 Fig Gene targeting and genotyping. (**A**) Detailed overview of the Tagger transgene and gene targeting. Arrows correspond to PCR primers used for genotyping. (**B**, **C**, **D**, **E**) Genotyping results using primers depicted in A. (**B**) PCR across homology regions; (**C**) PCR across the fragment containing STOP cassettes on DNA from not recombined (FL-Tagger), Flp-recombined (LSL-Tagger), and active Tagger; (**D**) PCR for the vector backbone (validation that no vector backbone integrated during gene targeting); (E) routine genotyping assay on homozygous (TAG/TAG) and heterozygous (TAG/+) Tagger; +/+–wild type. Ago2, Argonaute2; bGHpA, bovine growth hormone polyadenylation signal; CAG, chicken β-actin/CMV enhancer promoter element; CMV, cytomegalovirus; FL, FNF+LSL; FLAG, FLAG epitope tag; Flp, flippase; FNF, FRT-NeoR-FRT STOP cassette; HA, hemagglutinin tag; LSL, LoxP-STOP-LoxP cassette; RFP, red fluorescent protein; NC, negative control; NLS, nuclear localization signal; PC, positive control; P2A, Porcine Teschovirus 2A peptide; RISC, RNA-induced silencing complex; Rpl22, ribosomal protein 22; TgUPRT, *T*. *gondii* uracil phosphoribosyltransferase; V5, V5 epitope tag; WPRE, Woodchuck Hepatitis Virus Postranscriptional Response Element; 2A, P2A self-processing peptide.
(PNG)

S2 Fig Validation of Tagger expression in tissues and cells by multiple methods. (**A**) Expression of Tagger in different tissues assessed by immunoblot, with anti-FLAG antibody detecting the terminal component of the construct (Ago-Tag protein). Expression was detected in multiple heterozygous (TAG/+) but not WT (+/+) tissues. (**B**) Expression of Tagger in mouse retina, stained for V5 (Ago-Tag) in light purple. Dark brown tissue to the left is naturally pigmented epithelial cells. (**C**) FFPE sections from original Ribo-Tag mice (top) and Tagger mice (middle) immunostained for Rpl22 (HA); prefix “Omni-” refers to the ubiquitous activation of the transgenes; FNF-Tagger (bottom) was used as a control. Overall expression between the two mouse lines was indistinguishable. (**D**) Immunoblot comparing expression levels of Rpl22-HA protein in Tagger and in the original RiboTag mice; prefix “Omni-” refers to the ubiquitous activation of the transgenes. (**E**) Immunofluorescence verification of expression specificity, using antibodies directed to cell type marker proteins (parvalbumin, Gad67, and Satb2 for respectively PV, Gad2, and vGluT2 Taggers. RFP channel is endogenous fluorescence. (**F**) FACS analysis of natively isolated (unfixed) nuclei sorted using anti-HA antibodies (detecting Ribo-Tag protein). Only the NeuN-positive population was shown. FACS, fluorescence activated cell sorting; FFPE, formalin fixed paraffin embedded; FNF, FRT-NeoR-FRT; Gad2, glutamic acid decarboxylase 2; Gad67, glutamic acid decarboxylase 67; HA, hemagglutinin; NeuN, neuronal nuclei; PV, parvalbumin; RFP, red fluorescent protein; Rpl22, ribosomal protein 22; Satb2, special AT-rich sequence-binding protein 2; SSC, side scatter; vGluT2, vesicular glutamate transporter 2; WT, wild-type.
(PNG)

S3 Fig In vivo two-photon imaging. (**A**) Fluorescence of RFP-NLS expressing nuclei in the cortex at different depths of an anesthetized mouse. (**B**) Experimental time line for monitoring RFP-NLS expression using two-photon microscopy: imaging started directly after the cranial window surgery, allowing unilateral access to the somatosensory cortex. Expression of RFP-NLS was monitored immediately before and after a laser lesion for 240 minutes (every 5 minutes for 1 hour, every 10 minutes for 2 hours, and every 20 minutes for 1 hour). (**C**) Images depicting RFP-NLS expression of the same population of nuclei before, immediately after (0, 5, 10 minutes), and long after (60, 120, 180, 240 minutes) laser lesion (red circle). Images are maximum intensity projections (MIPs) of two z-sections with 3-μm z-steps and were acquired at a depth of 180 μm. White circle surrounds the region of decreased RFP-NLS expression surrounding the lesion, which increased in diameter during the 240 minutes after lesion. (**D**) Enlarged excerpt of B (inset at 240 minutes) showing the fate of RFP-expressing nuclei close to the site of lesion. Most of the nuclei kept their fluorescence (white arrow), while just a few nuclei showed a compartmentalization (violet arrow). MIP, maximum intensity projection; RFP-NLS, red fluorescent protein-nuclear localization signal.
(TIF)

S4 Fig Optimization of combined Ribo-Tag and Ago-Tag procedures. (**A**) I–III, Agilent bioanalyzer small RNA analysis of IP done with anti-HA (I), and anti-V5 (II) and anti-FLAG (III) antibodies. Antibodies directed to Ago-Tag (V5 and FLAG) lead to the retention of miRNAs (marked with arrows). The anti-HA antibody leads to much higher levels of other types of small RNAs included in the ribosomal complexes (small ribosomal RNA, tRNA, residual pre-miRNA species, etc.) but retains no detectable mature miRNAs. IV, Comparison of Ago-Tag IP done with anti-FLAG magnetic agarose (solid line trace) and a combination of anti-FLAG antibody and PGDB (gray filled trace) shows no noticeable difference in performance. (**B**) Ribo-Tag IP done with increasing stringency of wash buffer. (**C**) IP western blot of ribosomes and RISC. Rps6 and Ago2 coprecipitating with Rpl22-HA (top blot) and Dicer 1 and endogenous Ago2 coprecipitating with FLAG-V5-Ago2 (bottom blot). Top and bottom blots represent two different membranes loaded with equal amounts of the same samples. Ago2, Argonaute 2; HA, hemagglutinin; IP, immunoprecipitation; miRNA, microRNA; PGDB, Protein G Dynabeads; RISC, RNA-induced silencing complex; Rpl22, large subunit ribosomal protein 22; Rps6, small subunit ribosomal protein 6.
(TIF)

S5 Fig TU-Tag reproducibility. (**A**) Comparison of two independent purifications using TU-Tag. Heatmap is of Z-score calculated from TPM values, calculated as follows: Z = (x–mean(input))/SD(row), where SD is standard deviation. The complete set of TPM values related to batch 1 in A can be found in S1 Data. (**B**) The same experimental batches in A compared by PCA. Even though there is a batch effect, the variation between cell types is larger than the observed variation between batches. PCA, principal component analysis; TPM, transcripts per million.
(TIF)

S6 Fig Juxtaposition of Ribo-Tag, TU-Tag, and Nuc-Tag. For each method, bars represent LFC in Gad2 compared with vGluT2 samples. Only genes with |LFC| > 1 and FDR < 0.1 in all methods were shown. FDR, false discovery rate; Gad2, glutamic acid decarboxylase 2; LFC, Log_2_ fold change; vGluT2, vesicular glutamate transporter 2.
(TIF)

S1 Table Cell type–enriched mRNAs used to validate the Ribo-Tag component of the Tagger. The list was curated on the basis of the data obtained with the original RiboTag line (named with no hyphen, whereas in Tagger the related name contains a hyphen), and the obtained enriched genes fulfilling the criteria were post hoc validated for being previously reported as cell type–specific/marker genes. We sought a list of cell type–enriched or–depleted genes based on RiboTag data we produced for a separate project. Genes were chosen based on relative abundance in RNA samples captured from vGlut2-, Gad2-, PV- and Cx43-Cre–activated RiboTag hemibrains, and the specific criteria mentioned above the table. The result was that genes must be enriched or depleted in the context of whole brain samples to function as markers. Many known markers are specific only in certain regions. For example, Calbindin2 is specific to glutamatergic neurons in the cerebellum but is also expressed in GABAergic neurons in other brain regions, so it does not qualify as a marker in our list. Several of the genes qualifying as markers were already known markers or have been demonstrated by others to be cell type specific. This list was only used as a means to examine the efficiency of Tagger. Cre, causes recombination; Cx43, connexin 43; Gad2, glutamic acid decarboxylase 2; PV, parvalbumin; vGlut2, vesicular glutamate transporter 2.
(XLSX)

S2 Table Small RNAseq mapping statistics. Percentages of Oasis 2 reads mapping [28] to different small RNA biotypes. The between 10- and 30-fold increase in miRNA mappings in Ago-Tag samples (as compared with input) confirms that these samples are substantially enriched for miRNAs. miRNA, microRNA; piwiRNA, Piwi interacting RNA; RNAseq, RNA sequencing; rRNA, ribosomal RNA; snoRNA, small nucleolar RNA; snRNA, small nuclear RNA.
(XLSX)

S3 Table Results of statistical analysis of data related to Fig 5I. Dunn (1964) Kruskal-Wallis multiple comparison with *p*-values adjusted with the Benjamini-Hochberg method. All comparisons were significant.
(XLSX)

S4 Table Resources used in this study. (XLSX)

S1 Movie In vivo two-photon imaging of RFP-NLS expression in proximity of laser-lesioned brain tissue. RFP-NLS, red fluorescent protein-nuclear localization signal.
(AVI)

S1 Data Raw NGS data. NGS, next generation sequencing.
(XLSX)

## Abbreviations

Ago: Tag, Argonaute2 tag
Ago2: Argonaute 2
AU: arbitrary unit
CAG: cytomegalovirus:chicken actin fusion promoter
CaMKIIα: calcium/calmodulin dependent protein kinase II
Cre: causes recombination
DMR: differentially methylated region
EHSB: Extra High Salt Buffer
FACS: fluorescence activated cell sorting
FDR: false discovery rate
FFPE: formalin fixed paraffin embedded
Flp: flippase
FNF: Frt-NeoR-Frt
Frt: flippase recognition target
Gad2: glutamic acid decarboxylase 2
Gad67: glutamic acid decarboxylase 67
HA: hemagglutinin
HSB: High Salt Buffer
INTACT: isolation of nuclei tagged in specific cell types
IP: immunoprecipitation
IRES: internal ribosome entry site
LDS: lithium dodecyl sulfate
LFC: Log2 fold change
lncRNA: long noncoding RNA
LSL: LoxP-STOP-LoxP
MeDIP: methylated DNA immunoprecipitation
miRAP: miRNA affinity purification
miRNA: microRNA
mtDNA: mitochondrial DNA
MTSEA: methyl thiosulfonate ethylammonium
NeoR: neomycin resistance
NGS: next generation sequencing
NLS: nuclear localization signal
Nuc-Tag: nuclei tag
O/N: overnight
ORF: open reading frame
PCA: principal component analysis
PCL: principal cell layer of the hippocampus
PGDB: Protein G Dynabeads
PSB: Polysome Buffer
PV: parvalbumin
P2A: Porcine Teschovirus 2A peptide
RFP: red fluorescent protein
Ribo-Tag: ribosome tag; RISC
RNA: induced silencing complex
RNAseq: RNA sequencing
Rpl22: large subunit ribosomal protein 22
RPL22-HA: large subunit ribosomal protein 22-hemagglutinin (Ribo-Tag protein)
RPM: reads per million
Rps6: small subunit ribosomal protein 6
RT: room temperature
Satb2: special AT-rich sequence-binding protein 2
scRNAseq: single cell RNAseq
SILAC: stable isotope labeling with amino acids in cell culture
TES: transcription end site
TgUPRT: uracil phosphoribosyltransferase from *Toxoplasma gondii*
TK: thymidine kinase
TPM: transcripts per million
TRAP: translating ribosomes affinity purification
tRFP: turbo red fluorescent protein
TSS: transcription start site
TU-Tag: 4-thiouracil tag
vGluT2: vesicular glutamate transporter 2
VST: variance stabilizing transformation
WPRE: Woodchuck Hepatitis Virus Postranscriptional Response Element
WT: wild-type
4-TU: 4-thiouracil
