# Correction: Tagger—A Swiss army knife for multiomics to dissect cell type–specific mechanisms of gene expression in mice

**DOI:** 10.1371/journal.pbio.3001882

**Published:** 2022-11-01

**Authors:** Lech Kaczmarczyk, Vikas Bansal, Ashish Rajput, Raza-ur Rahman, Wiesław Krzyżak, Joachim Degen, Stefanie Poll, Martin Fuhrmann, Stefan Bonn, Walker Scot Jackson

Tagger mice in the C57Bl/6N background with the FNF cassette removed but the LSL cassette still in place (i.e., B6-LSL-Tagger) are now archived at the European Mouse Mutant Archive with the ID EM:14660: https://www.infrafrontier.eu/search?keyword=EM%3A14660.
