# Supplementary figures and images for: Tagger—A Swiss army knife for multiomics to dissect cell type–specific mechanisms of gene expression in mice
Source: PLoS Biol. 2019 Aug 8;17(8):e3000374. doi: 10.1371/journal.pbio.3000374 (PMC6701817; doi:10.1371/journal.pbio.3000374)

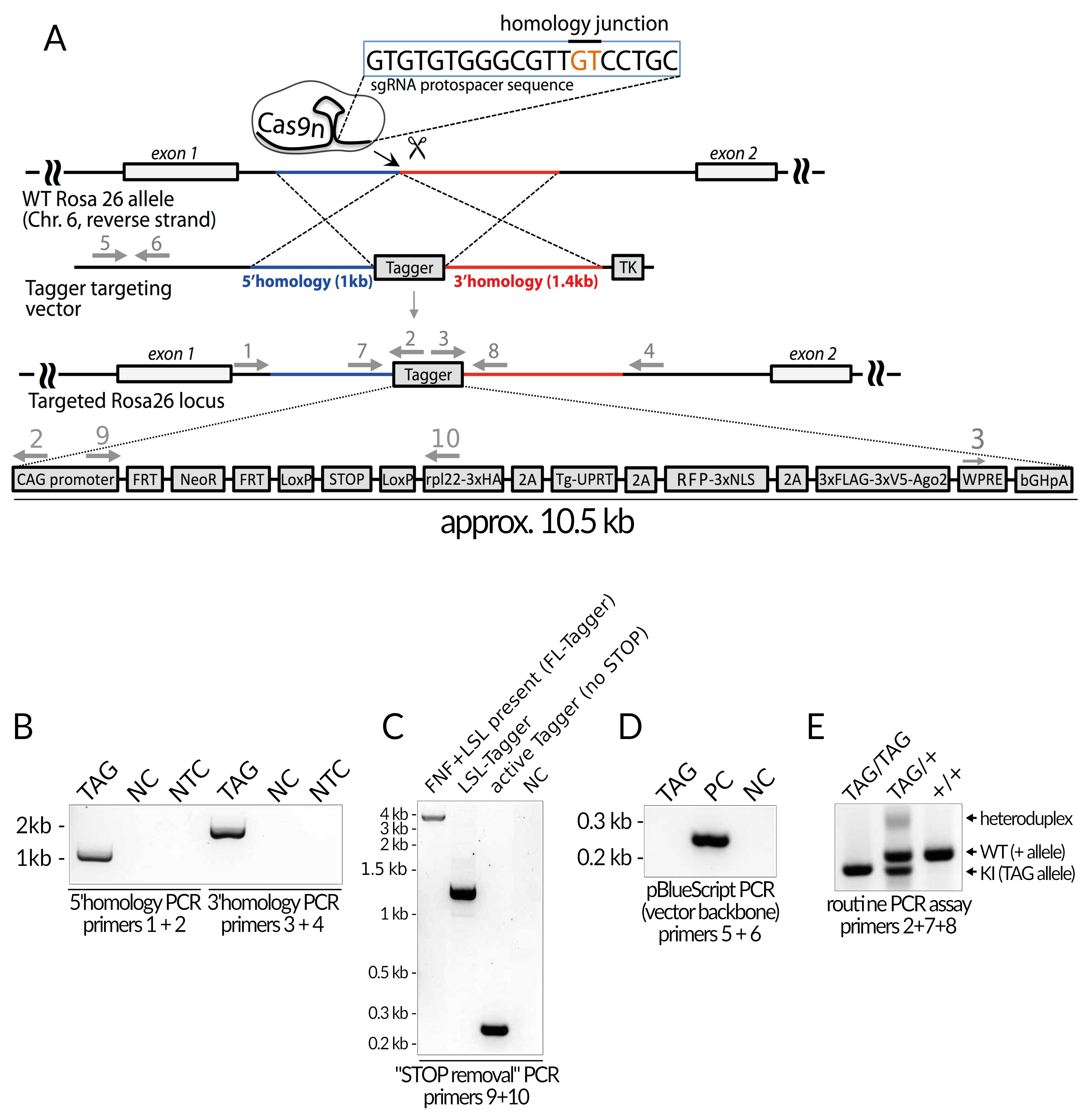

Supplement: S1 Fig — (A) Detailed overview of the Tagger transgene and gene targeting. Arrows correspond to PCR primers used for genotyping. (B, C, D, E) Genotyping results using primers depicted in A. (B) PCR across homology regions; (C) PCR across the fragment containing STOP cassettes on DNA from not recombined (FL-Tagger), Flp-recombined (LSL-Tagger), and active Tagger; (D) PCR for the vector backbone (validation that no vector backbone integrated during gene targeting); (E) routine genotyping assay on homozygous (TAG/TAG) and heterozygous (TAG/+) Tagger; +/+–wild type. Ago2, Argonaute2; bGHpA, bovine growth hormone polyadenylation signal; CAG, chicken β-actin/CMV enhancer promoter element; CMV, cytomegalovirus; FL, FNF+LSL; FLAG, FLAG epitope tag; Flp, flippase; FNF, FRT-NeoR-FRT STOP cassette; HA, hemagglutinin tag; LSL, LoxP-STOP-LoxP cassette; RFP, red fluorescent protein; NC, negative control; NLS, nuclear localization signal; PC, positive control; P2A, Porcine Teschovirus 2A peptide; RISC, RNA-induced silencing complex; Rpl22, ribosomal protein 22; TgUPRT, T. gondii uracil phosphoribosyltransferase; V5, V5 epitope tag; WPRE, Woodchuck Hepatitis Virus Postranscriptional Response Element; 2A, P2A self-processing peptide. (PNG) [file pbio.3000374.s001.png]

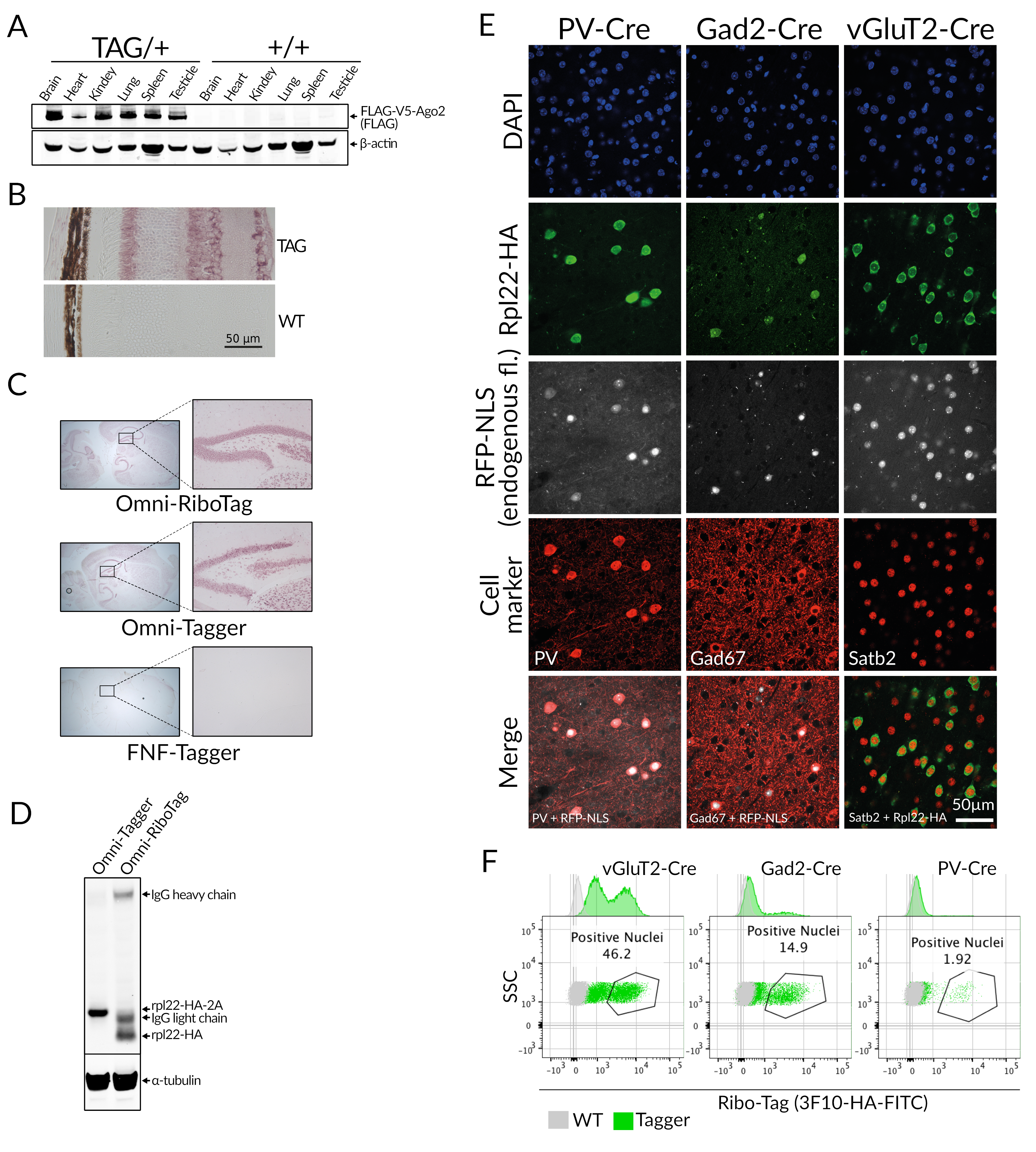

Supplement: S2 Fig — (A) Expression of Tagger in different tissues assessed by immunoblot, with anti-FLAG antibody detecting the terminal component of the construct (Ago-Tag protein). Expression was detected in multiple heterozygous (TAG/+) but not WT (+/+) tissues. (B) Expression of Tagger in mouse retina, stained for V5 (Ago-Tag) in light purple. Dark brown tissue to the left is naturally pigmented epithelial cells. (C) FFPE sections from original Ribo-Tag mice (top) and Tagger mice (middle) immunostained for Rpl22 (HA); prefix “Omni-” refers to the ubiquitous activation of the transgenes; FNF-Tagger (bottom) was used as a control. Overall expression between the two mouse lines was indistinguishable. (D) Immunoblot comparing expression levels of Rpl22-HA protein in Tagger and in the original RiboTag mice; prefix “Omni-” refers to the ubiquitous activation of the transgenes. (E) Immunofluorescence verification of expression specificity, using antibodies directed to cell type marker proteins (parvalbumin, Gad67, and Satb2 for respectively PV, Gad2, and vGluT2 Taggers. RFP channel is endogenous fluorescence. (F) FACS analysis of natively isolated (unfixed) nuclei sorted using anti-HA antibodies (detecting Ribo-Tag protein). Only the NeuN-positive population was shown. FACS, fluorescence activated cell sorting; FFPE, formalin fixed paraffin embedded; FNF, FRT-NeoR-FRT; Gad2, glutamic acid decarboxylase 2; Gad67, glutamic acid decarboxylase 67; HA, hemagglutinin; NeuN, neuronal nuclei; PV, parvalbumin; RFP, red fluorescent protein; Rpl22, ribosomal protein 22; Satb2, special AT-rich sequence-binding protein 2; SSC, side scatter; vGluT2, vesicular glutamate transporter 2; WT, wild-type. (PNG) [file pbio.3000374.s002.png]

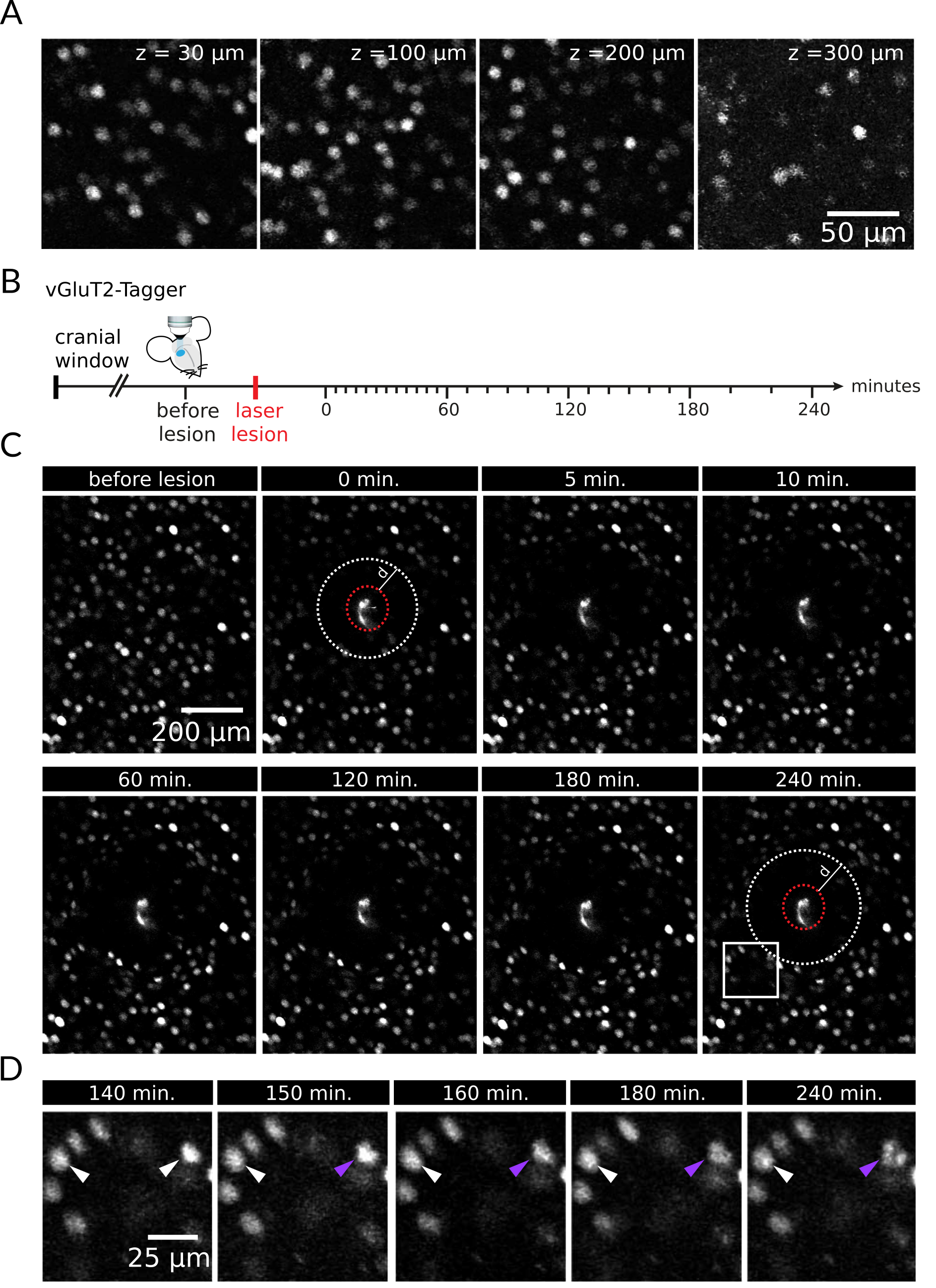

Supplement: S3 Fig — (A) Fluorescence of RFP-NLS expressing nuclei in the cortex at different depths of an anesthetized mouse. (B) Experimental time line for monitoring RFP-NLS expression using two-photon microscopy: imaging started directly after the cranial window surgery, allowing unilateral access to the somatosensory cortex. Expression of RFP-NLS was monitored immediately before and after a laser lesion for 240 minutes (every 5 minutes for 1 hour, every 10 minutes for 2 hours, and every 20 minutes for 1 hour). (C) Images depicting RFP-NLS expression of the same population of nuclei before, immediately after (0, 5, 10 minutes), and long after (60, 120, 180, 240 minutes) laser lesion (red circle). Images are maximum intensity projections (MIPs) of two z-sections with 3-μm z-steps and were acquired at a depth of 180 μm. White circle surrounds the region of decreased RFP-NLS expression surrounding the lesion, which increased in diameter during the 240 minutes after lesion. (D) Enlarged excerpt of B (inset at 240 minutes) showing the fate of RFP-expressing nuclei close to the site of lesion. Most of the nuclei kept their fluorescence (white arrow), while just a few nuclei showed a compartmentalization (violet arrow). MIP, maximum intensity projection; RFP-NLS, red fluorescent protein-nuclear localization signal. (TIF) [file pbio.3000374.s003.tif]

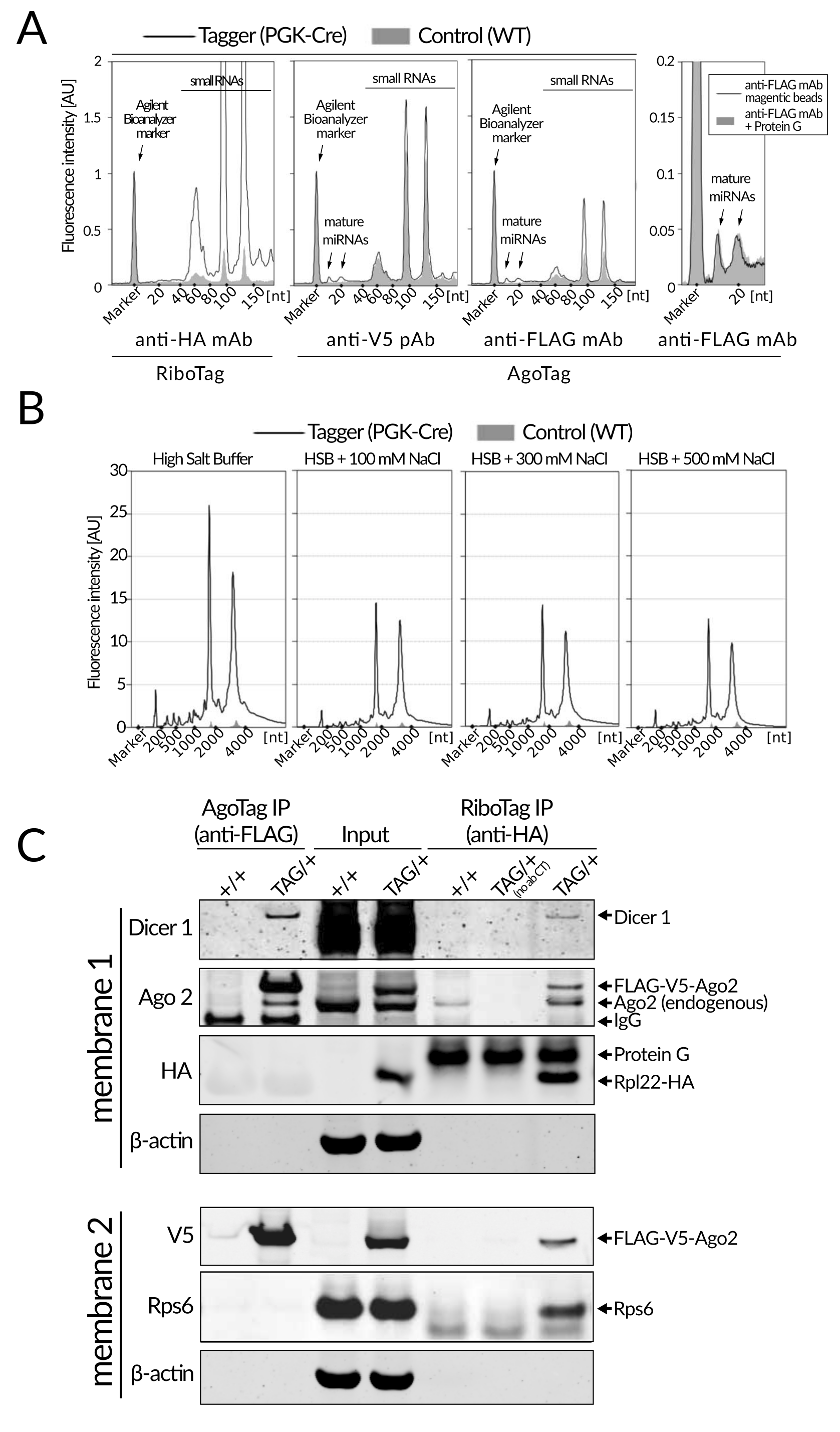

Supplement: S4 Fig — (A) I–III, Agilent bioanalyzer small RNA analysis of IP done with anti-HA (I), and anti-V5 (II) and anti-FLAG (III) antibodies. Antibodies directed to Ago-Tag (V5 and FLAG) lead to the retention of miRNAs (marked with arrows). The anti-HA antibody leads to much higher levels of other types of small RNAs included in the ribosomal complexes (small ribosomal RNA, tRNA, residual pre-miRNA species, etc.) but retains no detectable mature miRNAs. IV, Comparison of Ago-Tag IP done with anti-FLAG magnetic agarose (solid line trace) and a combination of anti-FLAG antibody and PGDB (gray filled trace) shows no noticeable difference in performance. (B) Ribo-Tag IP done with increasing stringency of wash buffer. (C) IP western blot of ribosomes and RISC. Rps6 and Ago2 coprecipitating with Rpl22-HA (top blot) and Dicer 1 and endogenous Ago2 coprecipitating with FLAG-V5-Ago2 (bottom blot). Top and bottom blots represent two different membranes loaded with equal amounts of the same samples. Ago2, Argonaute 2; HA, hemagglutinin; IP, immunoprecipitation; miRNA, microRNA; PGDB, Protein G Dynabeads; RISC, RNA-induced silencing complex; Rpl22, large subunit ribosomal protein 22; Rps6, small subunit ribosomal protein 6. (TIF) [file pbio.3000374.s004.tif]

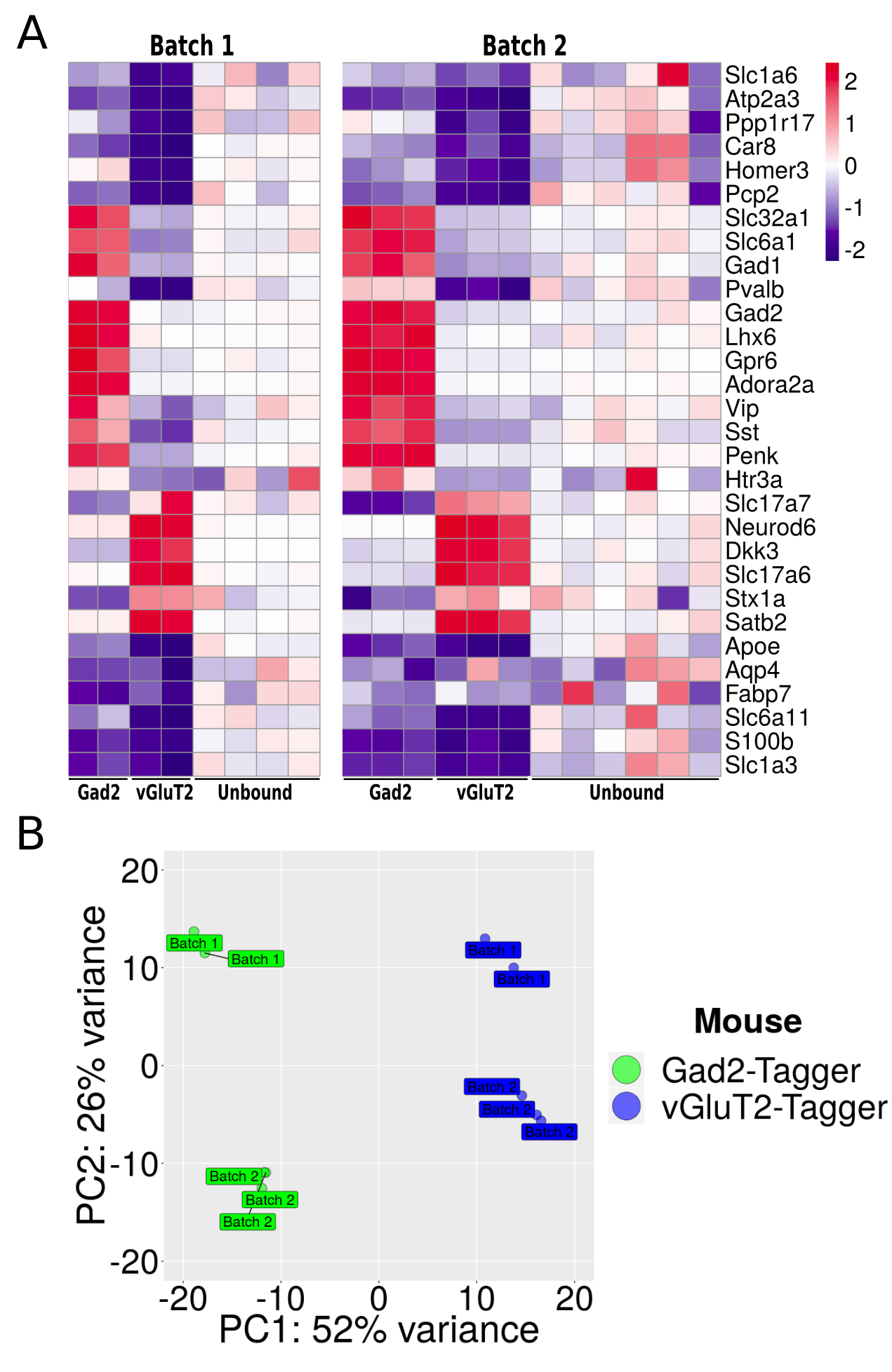

Supplement: S5 Fig — (A) Comparison of two independent purifications using TU-Tag. Heatmap is of Z-score calculated from TPM values, calculated as follows: Z = (x–mean(input))/SD(row), where SD is standard deviation. The complete set of TPM values related to batch 1 in A can be found in S1 Data. (B) The same experimental batches in A compared by PCA. Even though there is a batch effect, the variation between cell types is larger than the observed variation between batches. PCA, principal component analysis; TPM, transcripts per million. (TIF) [file pbio.3000374.s005.tif]

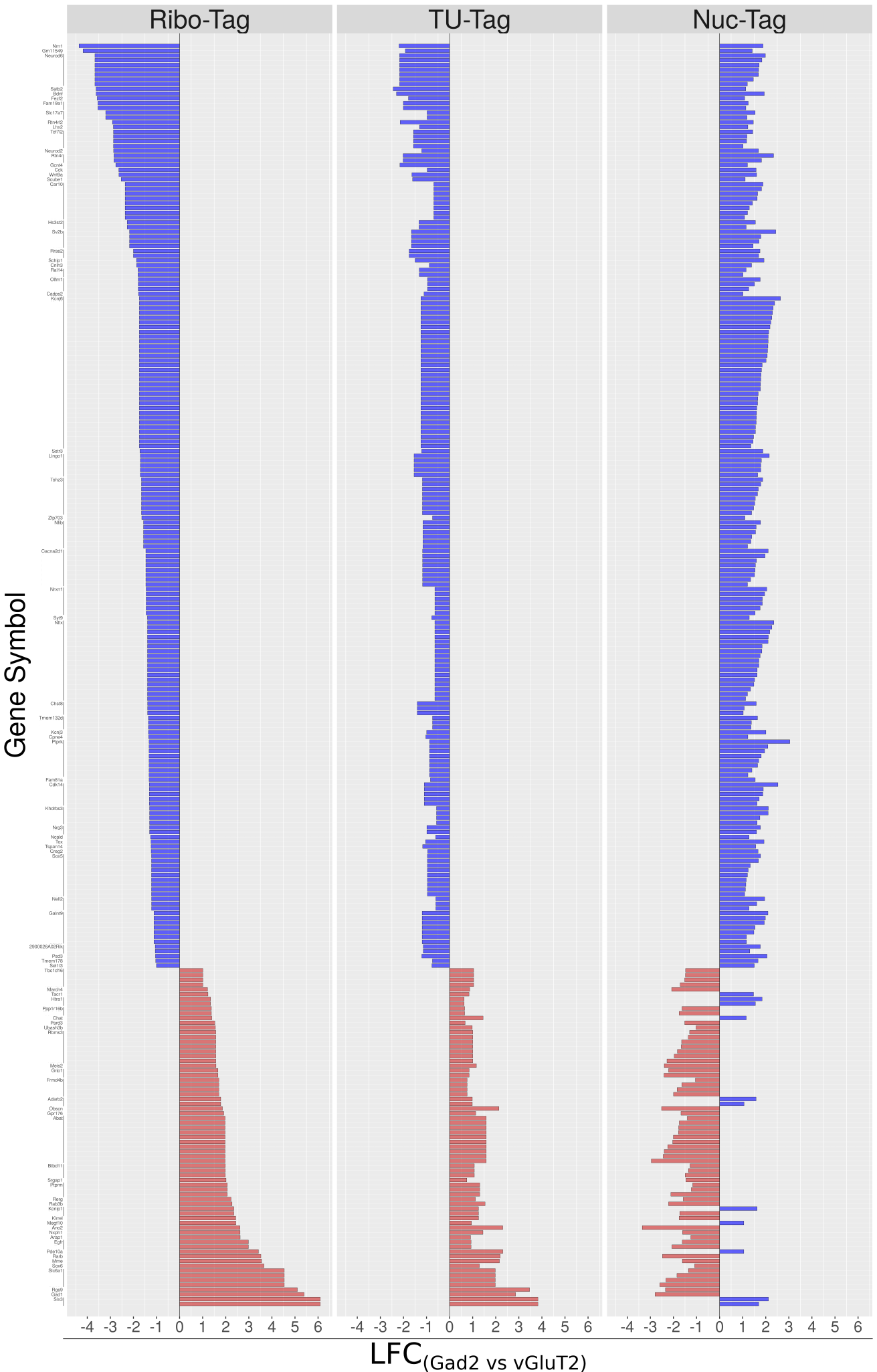

Supplement: S6 Fig — For each method, bars represent LFC in Gad2 compared with vGluT2 samples. Only genes with |LFC| > 1 and FDR < 0.1 in all methods were shown. FDR, false discovery rate; Gad2, glutamic acid decarboxylase 2; LFC, Log2 fold change; vGluT2, vesicular glutamate transporter 2. (TIF) [file pbio.3000374.s006.tif]
